# Using host-pathogen protein interactions to identify and characterize *Francisella tularensis* virulence factors

**DOI:** 10.1186/s12864-015-2351-1

**Published:** 2015-12-29

**Authors:** Anders Wallqvist, Vesna Memišević, Nela Zavaljevski, Rembert Pieper, Seesandra V. Rajagopala, Keehwan Kwon, Chenggang Yu, Timothy A. Hoover, Jaques Reifman

**Affiliations:** Department of Defense Biotechnology High Performance Computing Software Applications Institute, Telemedicine and Advanced Technology Research Center, U.S. Army Medical Research and Materiel Command, Fort Detrick, MD 21702 USA; J. Craig Venter Institute, Rockville, MD 20850 USA; Bacteriology Division, U.S. Army Medical Research Institute of Infectious Diseases, Fort Detrick, MD 21702 USA

**Keywords:** Host-pathogen interactions, Protein-protein interactions, *Francisella tularensis*, Intracellular pathogen, Virulence factors

## Abstract

**Background:**

*Francisella tularensis* is a select bio-threat agent and one of the most virulent intracellular pathogens known, requiring just a few organisms to establish an infection. Although several virulence factors are known, we lack an understanding of virulence factors that act through host-pathogen protein interactions to promote infection. To address these issues in the highly infectious *F. tularensis* subsp. *tularensis* Schu S4 strain, we deployed a combined *in silico*, in vitro, and in vivo analysis to identify virulence factors and their interactions with host proteins to characterize bacterial infection mechanisms.

**Results:**

We initially used comparative genomics and literature to identify and select a set of 49 putative and known virulence factors for analysis. Each protein was then subjected to proteome-scale yeast two-hybrid (Y2H) screens with human and murine cDNA libraries to identify potential host-pathogen protein-protein interactions. Based on the bacterial protein interaction profile with both hosts, we selected seven novel putative virulence factors for mutant construction and animal validation experiments. We were able to create five transposon insertion mutants and used them in an intranasal BALB/c mouse challenge model to establish 50 % lethal dose estimates. Three of these, ΔFTT0482c, ΔFTT1538c, and ΔFTT1597, showed attenuation in lethality and can thus be considered novel *F. tularensis* virulence factors. The analysis of the accompanying Y2H data identified intracellular protein trafficking between the early endosome to the late endosome as an important component in virulence attenuation for these virulence factors. Furthermore, we also used the Y2H data to investigate host protein binding of two known virulence factors, showing that direct protein binding was a component in the modulation of the inflammatory response via activation of mitogen-activated protein kinases and in the oxidative stress response.

**Conclusions:**

Direct interactions with specific host proteins and the ability to influence interactions among host proteins are important components for *F. tularensis* to avoid host-cell defense mechanisms and successfully establish an infection. Although direct host-pathogen protein-protein binding is only one aspect of *Francisella* virulence, it is a critical component in directly manipulating and interfering with cellular processes in the host cell.

**Electronic supplementary material:**

The online version of this article (doi:10.1186/s12864-015-2351-1) contains supplementary material, which is available to authorized users.

## Background

*Francisella tularensis*, the causative agent of tularemia and a Centers for Disease Control and Prevention Category A select agent, is one of the most infectious bacteria known, with <10 cells capable of causing severe infections in humans and animals [1]. Its role as a biological weapon has been investigated in the United States (U.S.), the former Soviet Union, and Japan [2], and it remains both a potential health hazard and a bioterrorism threat. *F. tularensis* is a Gram-negative opportunistic intracellular pathogen that primarily affects lagomorphs but can infect virtually any human cell type, although the primary infection typically goes through macrophages [3, 4]. As such, *F. tularensis* is endowed with multiple robust infection mechanisms, including the ability to avoid or circumvent host immune defenses [5–11].

Relatively little is known about the detailed molecular nature of *F. tularensis* pathogenicity, although recent progress in high-throughput genomic and proteomic technologies has contributed to an increase in the number of experimentally confirmed or predicted genes involved in its virulence [12]. By definition, inactivation of these “virulence factors” attenuates or abrogates virulence in an animal infection model, regardless of which stages of the infection process they are involved in. Given the high virulence and large number of identified virulence factors for this organism, one can surmise that multiple robust mechanisms are contributing to the infection process.

*Francisella* spp. virulence is highly correlated with the presence of the so-called pathogenicity islands among the most pathogenic strains and their modification or absence in non-pathogenic strains. A 33-kbp pathogenicity island has been identified in *F. tularensis* [13], and systematic mutations confirmed its key role in virulence [14]. A region of the pathogenicity island encodes a set of genes that map to a fully functional type VI secretion system (T6SS) [15, 16]. In *F. tularensis*, T6SSs play a central role in manipulating host cells by translocating virulence factors across the bacterium’s inner and outer membranes directly into the host cytosol. Alternatively, bacterial proteins can also be exported to the periplasmic space via the universal Sec pathway and then translocated across the outer membrane, usually via a type II secretion system (T2SS) [17, 18]. The identity of the specific proteins translocated through these systems and their interactions with host proteins or other factors in the host cell are not well characterized. However, host-pathogen interactions provide a means to identify virulence factors that are directly related to protein interactions and ascertain their associated mechanisms of infectivity [19, 20].

Accordingly, we deployed a bioinformatics-based approach to initially select *F. tularensis* subsp. *tularensis* Schu S4 proteins broadly associated with secretion and filtered the selected putative virulence factors through whole-genome human and murine yeast two-hybrid (Y2H) screens to select unstudied bacterial proteins that exhibited host-pathogen protein-protein interactions (PPIs). Seven of these bacterial proteins were selected for transposon mutant construction, five of which were successfully obtained and used in an intranasal BALB/c mouse challenge model to ascertain in vivo virulence. Three of these, ΔFTT0482c, ΔFTT1538c, and ΔFTT1597, showed a statistically significant reduction in lethality compared with the wild-type strain and can, thus, be considered novel virulence factors. Based on the accompanying Y2H data, we could also generate hypotheses about the underlying host mechanisms targeted by infecting bacteria.

## Methods

### Bioinformatics and literature-based identification of potential virulence factor proteins

We initially identified and collected putative *Francisella* spp. virulence factor proteins based on comparative genomics between high- and low-pathogenicity strains, the presence of VgrG domains as indicative of T6SS association [21], model predictions of signaling sequences, and literature-based searches. We obtained all *Francisella* genomes from the PathoSystems Resource Integration Center database [22].

Using QuartetS [23, 24], we performed whole-genome comparisons of six highly pathogenic *F. tularensis* strains with eight less pathogenic *F. tularensis* strains and seven *F. novicida* strains (Additional file 1: Table S1) to identify proteins present only in the highly pathogenic strains. To identify putative VgrG proteins, we initially downloaded VgrG domain-containing genes from the National Center for Biotechnology Information (NCBI) database [25]. Using BLAST [26], we compared the *F. tularensis* subsp. *tularensis* Schu S4 genome with all other VgrG domain-containing genes and identified putative *F. tularensis* VgrG genes based on homology. We considered two genes as homologs if the E-value of their alignment was ≤0.01.

We used SignalP to predict the presence and location of signal peptide cleavage sites in amino acid sequences of the *F. tularensis* subsp. *tularensis* Schu S4 genome [27]. In prokaryotes, these signal peptides constitute ubiquitous protein-sorting signals that target their passenger proteins for translocation across the cytoplasmic membrane, although the presence of the signal peptide does not guarantee that a protein is secreted. We used both the hidden Markov model method and the neural network method in SignalP to identify *F. tularensis* proteins with signal peptides to tag them as putatively secreted proteins. We used default cutoff values for both methods and designated putative signaling proteins as those proteins identified using both methods.

### High-throughput Y2H screens to identify human-*F. tularensis* PPIs

We cloned the known and predicted *F. tularensis* virulence factor genes from *F. tularensis* subsp. *tularensis* Schu S4 genomic DNA. All *F. tularensis* virulence factor genes were PCR amplified using gene-specific primers incorporated with forward and reverse Gateway recombination cloning sequences, i.e., attB1 (5’-GGGGACAAGTTTGTACAAAAAAGCAGGCTTC-3’) and attB2 (5’-GGGGACCACTTTGTACAAGAAAGCTGGGTC-3’), respectively. The PCR-amplified open reading frames (ORFs) were cloned into Gateway entry vector pDONR221™ as recommended by the BP Clonase™ II enzyme provider (Invitrogen, Carlsbad, CA). We validated the entry vectors with the cloned ORFs by Sanger sequencing.

Entry clones were sub-cloned into Y2H bait (DNA-binding domain) vectors, pGBGT7g (as NH_2_-terminal fusion) and pGBACg (C-terminal fusion) [28], using Gateway LR reactions (Invitrogen). Y2H bait clones were subsequently transferred into the haploid yeast strain AH109 (MAT-α), as previously described [29]. Before the two-hybrid analyses, we examined all baits in yeast strains for auto-activation, i.e., detectable bait-dependent reporter gene activation in the absence of any interacting protein. We used the HIS3 reporter gene, and auto-activation was titrated by adding different concentrations of 3-amino-1,2,4-triazole (3-AT), a competitive inhibitor of HIS3. All *F. tularensis* baits were inspected for auto-activation on plates containing different concentrations of 3-AT. The lowest concentration of 3-AT that suppressed growth for the interaction screen was used because it avoided background growth while still detecting true interactions.

A haploid yeast strain expressing each *F. tularensis* protein as bait was used for the PPI screening with human and mouse normalized universal cDNA libraries (catalog nos. 630480 and 630482, Clontech Laboratories, Mountain View, CA). The bait and prey yeast culture was grown and mixed at a 1:1 ratio and plated on yeast extract, peptone, dextrose, and adenine (YEPDA) agar plates. YEPDA agar plates were incubated at 30 °C for 6 h or overnight at room temperature. During this process, both prey and bait plasmids were combined in diploid yeast cells by yeast mating. Cells from the mating plates were collected and transferred onto interaction-selection yeast-synthetic medium with predefined concentrations of 3-AT (media lacking tryptophan, leucine, and histidine plus 3-AT), and plates were incubated at 30 °C for 4–6 days. The interaction-selection plates that showed colony growth but no colonies on control plates (bait mated to empty prey vector) were identified as two-hybrid positive yeast clones. Positive yeast colonies were selected manually and subjected to yeast colony PCR followed by DNA sequencing to identify the interacting preys [29].

### Pairwise Y2H retesting of human-*F. tularensis* high-throughput PPIs

To increase confidence in selected human-*F. tularensis* virulence factor protein interactions, we designated 12 interaction pairs identified in the high-throughput Y2H library screening for retesting in pairwise Y2H assays. Human prey clones were constructed by sub-cloning the ORFs from the Human ORFeome collection [30] or amplifying the ORF from the Mammalian Gene Collection library [31] and subsequently transferred into the two Y2H prey vectors (pGADT7g and pGBACg). The pairwise Y2H assays were performed with activation tests conducted side by side as previously described [29].

### Generation of transposon insertion mutants

The *F. tularensis* subsp. *tularensis* Schu S4 strain used in this study was derived from a U.S. Army Medical Research Institute for Infectious Diseases (USAMRIID) stock of an *F. tularensis* isolate obtained from a U.S. patient in 2005 and supplied by McKesson BioServices (Rockville, MD). A seed stock of that strain was passaged twice through mice, and single colony isolates were collected, pooled, and labeled FT12. In these experiments, in vitro culturing of FT12 was done at 37 °C in enhanced tularemia broth (ETB) consisting of trypticase soy broth (Becton Dickinson, Franklin Lakes, NJ) supplemented with cysteine (0.1 %), glucose (0.1 %), ferric pyrophosphate (0.25 %), and horse serum (2.5 %). Growth of FT12 on solid medium was done on enhanced tularemia agar (ETA) plates consisting of Mueller-Hinton II agar (Becton Dickinson) supplemented with NaCl (2.5 g/l), protease peptone (5 g/l), cysteine (0.1 %), glucose (0.1 %), ferric pyrophosphate (0.25 %), and horse serum (2.5 %).

Plasmid pGreenhopper (T.A. Hoover, unpublished data) was constructed in plasmid pCR2.1-TOPO (Life Technologies, Frederick, MD) and carries a modified Mariner-based Himar 1 transposon and flanking transposase gene. Within the inverted DNA repeats that define the termini of the transposon is a kanamycin resistance gene positioned downstream of the promoter for the FT12 groESL operon to allow for selection of cells harboring transposon insertions. pGreenhopper was electroporated into FT12, diluted, and plated on 20 ETA plates. Approximately 2,000 colonies per plate were scraped and pooled for use in high-throughput mutagenesis screening experiments. Southern blot analysis of 30 independent *F. tularensis* transposon recipients suggested that the library was random and that transposition events occurred once per cell, presumably due to the lack of replication of pGreenhopper in *F. tularensis* and the conservative cut-and-paste mode of replication characteristic of Mariner family transposable elements.

Specific *F. tularensis* Schu S4 mutants were isolated from the pooled collection of approximately 40,000 independent transposon insertion mutants by nested PCR detection with progressively smaller library subsets. Primers that annealed downstream of a gene of interest were paired with primers that annealed to the terminal repeats of the inserted transposon in PCRs containing small aliquots of whole cells from subsets of the *F. tularensis* library (Table 1). Subsets giving rise to appropriately sized DNA bands from these reactions were cultured on ETA plates to obtain smaller subsets, and the process was repeated until single colony isolates were obtained and stored at -80 °C. Genomic DNAs were prepared from these clones and re-analyzed by PCR to confirm the presence of the transposon within the coding sequence of each gene. In two cases (FTT1538c and FTT1564), insertion sites were determined by DNA sequencing.

**Table 1:** Primers, plasmids, and strains used in the mouse intranasal challenge model experiments

Primer, plasmid, or strain	Relevant characteristics	Source or reference
Primer		
FTT0482d5’	GCTCAACATTGTATGAAATTAATGGCTCCA	Invitrogen
FTT0482p5’	GTGAAATAGTCAGACAAGTAAGCCTTGGT	Invitrogen
FTT0482d3’	ATTTGTATCAGCCAAATGCTGTTACGCA	Invitrogen
FTT0482p3’	GTAGAATGTGGATGAATGTTAAGTACGGT	Invitrogen
FTT0902d5’	CTGCTGCTGCTCAGACAGCTACTACTG	Invitrogen
FTT0902p5’	GCTGCTGCTGTATCTAAGCCAACTGC	Invitrogen
FTT1538d5’	CAGCAGGCGATTATGGCTACAAACA	Invitrogen
FTT1538p5’	GCAGGTCAGATGTCGACACAAGAAGC	Invitrogen
FTT1538d3’	TGCTTCAGCTTCGGACTTAGCAACA	Invitrogen
FTT1538p3’	ATCCAATTGCTGCATCCACACCATCA	Invitrogen
FTT1564d5’	CTCCTCCTCATATCAGTTCTGTCAGCT	Invitrogen
FTT1564p5’	GTCGGTTTCCCAAGCTACTGGAATGT	Invitrogen
FTT1564d3’	GGACTCGAACCTACGACCTACGGAT	Invitrogen
FTT1564p3’	CTAACCAACTGAGCTATAGGCCCA	Invitrogen
FTT1597d5’	CGGCTGATAATGATGGCTTTATGGCT	Invitrogen
FTT1597p5’	TGATCCTCCTGAATATGATGATCCTAGT	Invitrogen
FTT1597d3’	GCATATACGGCTGAATCTTGCCACCT	Invitrogen
FTT1597p3’	CAATGATGCCAATGCCGCGGTAACT	Invitrogen
Plasmid		
pCR2.1-TOPO	3,931-bp TA vector; pMB1 *oriR*; Km^r^	Life Technologies
pGreenhopper	pCR2.1-TOPO containing a 4-kbp Mariner-Himar 1 transposon with kanamycin resistance gene and a 1.2 kbp tnpA transposition gene. Non-replicable in *F. tularensis*	Unpublished
*E. coli*		
TOP10	General cloning and blue/white screening	Life Technologies
*F. tularensis*		
FT12	Type strain, a mouse-passaged isolate of a strain obtained from a U.S. patient	McKesson BioServices

### Animal experimentation

BALB/c mice have been widely used and accepted for the experimental study of tularemia and was the animal model chosen for these experiments. Female BALB/c mice at 6–9 wk of age were obtained and intranasally administered doses of wild-type *F. tularensis* Schu S4 and selected mutants, and then observed for 21 days. Moribund mice and mice surviving to *day 21* post-challenge were euthanized in accordance with USAMRIID Standard Operating Procedure AC-11–04–05.

Challenge doses were prepared from overnight *F. tularensis* cultures grown at 37 °C on ETA plates. Cells were scraped and diluted in phosphate-buffered saline solution to obtain appropriate numbers of cells in each dose. Intranasal installations of 33 μl/mouse were followed by 10-μl washes in each nostril. Mice were anesthetized during this procedure by an intramuscular injection of 100 μl of ketamine-acepromazine-xylazine in accordance with USAMRIID Standard Operating Procedure AC-09–10.

This study was conducted in compliance with the Animal Welfare Act and other federal statutes and regulations relating to animals and experiments involving animals and with adherence to principles stated in the Guide for the Care and Use of Laboratory Animals (www.nap.edu/readingroom/books/labrats/chaps.html). The facility where this research was conducted is fully accredited by the Association for Assessment and Accreditation of Laboratory Animal Care International. The USAMRIID Institutional Animal Care and Use Committee reviewed and approved the animal protocol entitled “Pathogenesis studies of *F. tularensis* mutants in mice,” (Animal Protocol no. AP-11–013).

### Growth rate experiments

Select *F. tularensis* subsp. *tularensis* Schu S4 strain transposon insertion mutants were cultured in chemically defined Chamberlain’s growth medium, and their growth rates, as determined by optical density measurements at 600 nm (OD_600_), were compared to those of the parent strain. Frozen stocks of each mutant and the parent strain were thawed on ice and diluted into 2 ml growth media at an OD_600_ of ~0.150. Culture tubes were incubated at 37 °C while constrained in a slanted rack and shaken at 150 rpm. Cultures were sampled for OD_600_ readings as a function of time, and their values were recorded.

### Biosafety and biosecurity

The *Francisella* experiments were conducted in Biosafety Level 3 containment laboratories at USAMRIID with appropriate personal safety and biological select agents and toxins security measures in place. We do not anticipate that this report provides knowledge, products, or technologies that could be directly misapplied by others to pose a threat to public health and safety, agricultural crops and other plants, animals, the environment, or materiel. The Institutional Biosafety Committee (IBC) that approved this work is composed of members of the USAMRIID research staff, Commander’s office, and qualified representatives from external institutions and is tasked to provide local, institutional oversight of research using recombinant DNA. The USAMRIID IBC was established under the U.S. National Institutes of Health Guidelines for Research Involving Recombinant DNA Molecules.

### 50 % Lethal dose estimation

We used Bayesian probit analyses to estimate the lethal dose response. As a prior distribution, we used a weakly informative Cauchy distribution with center 0 and scale 10. Parameters were assumed independent of each other. Using samples from the posterior distribution and intercept parameters from the probit analysis, we estimated the 50 % lethal dose (LD_50_) and 95 % confidence intervals as well as the likelihoods of each strain being more or less potent than any other strain at the median lethality level.

### Survival time comparison

We used the Kaplan-Meier method to estimate the probability of animals surviving a given length of time [32] and a G-rho class of rank tests to compare difference between the obtained survival curves [33]. We calculated all probabilities using the R analysis package “survival” (Terry Therneau, “A Package for Survival Analysis in S,” R package version 2.37–7, 2014).

### Creation of the expanded human-*F. tularensis* PPI network

We used the NCBI HomoloGene database of homologs (www.ncbi.nlm.nih.gov/homologene) to identify human-murine orthologs [34]. These orthologs were further used to create sets of *1*) conserved PPIs between human-*F. tularensis* and murine-*F. tularensis* and *2*) expanded *F. tularensis*-human PPIs consisting of experimentally detected and orthology-based predicted protein interactions.

We identified the conserved set of PPIs as those PPIs where the human protein interacted with the same *F. tularensis* protein as their murine orthologs. To create the expanded set of *F. tularensis*-human PPIs we merged the human data with orthologous mouse data. To identify the orthologous data, we first identified the 11 *F. tularensis* proteins that had protein interactions with both hosts. Among these interactions, we identified the set of murine-*F. tularensis* PPIs for which there exists a human ortholog to the murine protein. The resulting orthologous data set consisted of 76 interactions between 74 human proteins and the 11 *F. tularensis* proteins, corresponding to 64 % of the murine-*F. tularensis* PPIs. Finally, we merged the human-*F. tularensis* experimental and orthologous data sets to create the expanded set of human-*F. tularensis* PPIs consisting of 298 unique interactions between 18 *F. tularensis* and 249 human proteins. These PPI data sets are provided in the supplementary information (Additional file 2: PPI data).

All networks were plotted in Cytoscape [35] using R packages iGraph and RCytoscape [36, 37].

### Gene Ontology annotation of host genes

We performed two types of Gene Ontology (GO) [38] enrichment analysis: standard enrichment analysis and network-based enrichment analysis. Both enrichment analyses were performed in R using the Bioconductor packages BioMart [39]. GO annotations were obtained from BioMart [39]. We used all annotation levels of the GO tree, excluding only terms associated with the root and top two levels. We used the Benjamini and Hochberg multiple test correction to account for multiple hypothesis testing [40].

In the standard enrichment analysis, we used the hypergeometric distribution to assess the statistical significance of observing a given GO term in our data. As the complement of human proteins, we used the set of all human/murine proteins available in BioMart [39] and annotated with at least one GO term. The reported results contain only GO terms that were enriched at a ≤20 % false discovery rate.

### Network analysis of host-pathogen PPIs: interaction modules

In the network-based enrichment analysis, we first identified connected (sub)networks of the largest connected component (LCC) in the human interactome that consists of human proteins interacting with at least one *F. tularensis* protein and in which all human proteins were annotated with the same GO term. We denote these (sub)networks as “interaction modules.” We then assessed the statistical significance of observing a given interaction module using Monte Carlo simulation. Specifically, we assessed probabilities of observing the same interaction module given *1*) a set of random human proteins of the same size as the set of human proteins interacting with *F. tularensis* and *2*) a set of random (randomly rewired) human PPIs, where the degree (number of interactions) of each protein was maintained to the same value as in the full set of human PPIs [20]. We kept only interaction modules consisting of four or more proteins. For the full set of human proteins, we used all constituent proteins from a human PPI network [41]. The reported results contain only GO terms that were enriched at a ≤5 % false discovery rate.

## Results

### *In silico* identification of potential virulence factors

We used multiple independent strategies to identify potential virulence factors of *F. tularensis* subsp. *tularensis* Schu S4. We initially used orthology to identify eight proteins present only in six genomes of highly pathogenic *F. tularensis* genomes and absent in eight less pathogenic *F. tularensis* and seven *F. novicida* genomes (Additional file 1: Table S1) [23, 24]. Given the importance of T6SSs in *Francisella* virulence [21], we further identified 16 putative proteins with homology to VgrG domain-containing proteins and, thus, potentially a component of the T6SS. We used SignalP to identify 290 proteins predicted to be secreted by the general secretory pathway. Finally, we examined the available literature on *Francisella* high-throughput and small-scale virulence screens to compile a list of 300 proteins associated with virulence [3–6, 12, 42–44].

### Selection of potential virulence factor proteins for Y2H experiments

To select proteins for experimental evaluation, we first merged the lists of potential virulence factors and down-selected proteins based on expert knowledge weighing the available evidence. As our focus was on detecting novel virulence factors, we initially excluded proteins previously studied in animal model experiments. From the list of predicted secreted proteins, we excluded 226 proteins *1*) lacking annotations and predicted to be secreted with a probability lower than 1.0 or *2*) proteins with annotations but predicted to be secreted with a probability of <0.9. From the list of 300 experimentally screened proteins, we excluded capsule proteins whose role in virulence is controversial [45], acid phosphatases as they have recently been proven less important for virulence [46], and metabolic proteins and bacterial regulators as these proteins are not likely to be secreted. We also excluded all membrane and transporter proteins except those that were identified from our comparative genomics or signal peptide analysis. Furthermore, we removed type IV pili proteins and proteins associated with *Francisella* pathogenicity islands encoding T6SS, as they have already been extensively studied in animal model experiments. The exceptions were type IV pili protein FTT1314c, predicted to be secreted by the general secretory pathway and putatively associated with the T2SS [47], and the T6SS proteins FTT1712c (IgIC2) and FTT1707 (IgIl2) that have been shown to be secreted in proteomics experiments [15, 48] and to contribute to pathogenicity [14, 16], but their host interacting partners are not known. Furthermore, we kept all proteins annotated as hypothetical or of unknown function, as long as their orthologs were present in the *F. tularensis* subsp. *tularensis* Schu S4 genome.

We further removed all proteins shorter than 90 amino acids and proteins annotated as essential in the Database of Essential Genes [49], as these latter proteins would prevent mutant creation. The resulting list of proteins contained 119 unique potential virulence factors. Of these, 97 had not previously been tested in murine models; 22 had been tested in murine models, but their interactions with host proteins are not known. We then ranked and grouped the set of 119 proteins into five groups based on the confidence level we could associate with each protein as a secreted virulence factor. We assigned proteins the highest level of confidence for those with multiple known virulence associations and evidence about attenuation in a mouse animal model from high-throughput screening of *F. tularensis* subsp. *tularensis* Schu S4 using the Transposon Directed Insertion Site Sequencing technique (T.A. Hoover, unpublished data). We assigned the lowest level of confidence to those proteins for which we only had orthology-based evidence.

Finally, we individually assessed each protein and down-selected the list into a final, pruned set of 49 putative and known virulence factors suitable for experimental evaluation (Table 2).

**Table 2:** List of proteins selected for experimental yeast two-hybrid evaluation

*Group*	Locus ID	Name	Description	Interactions		
				Human	Mouse	Common
*1*	FTT0369c	DipA	Deficient in intracellular replication A	-	-	-
	FTT0584	-	Unknown	-	-	-
	FTT0901	LpnA	Lipoprotein	-	1	-
	FTT0918	FopC	Outer membrane lipoprotein	-	-	-
	FTT1103	DsbA	Conserved hypothetical lipoprotein	-	-	-
	FTT1179	BipA	GTP binding translational elongation factor Tu	29	-	-
	FTT1508c	RelA	GTP pyrophosphokinase	3	-	-
	FTT1726	YegQ	Protease YegQ	-	-	-
	FTT1676	-	Protein of unknown function, membrane protein	-	-	-
	FTT0356	HtpG	Chaperone, heat shock protein HtpG, ATPase activity	20	1	1
	FTT0626	Lon	Chaperone, ATP-dependent DNA-binding protease Lon	2	2	-
	FTT1769c	ClpB	Chaperone, ATP-dependent CLP protease ATP-binding subunit ClpB	-	-	-
	FTT1712c	IglC2	Intracellular growth locus, subunit C	43	33	1
	FTT0068	SodB	Superoxide dismutase	-	12	-
	FTT1707	IglI2	Uncharacterized protein	-	-	-
*2*	FTT0013c	-	Protein of unknown function	-	-	-
	FTT0295	-	Hypothetical protein	-	-	-
	FTT0520	-	Putative uncharacterized protein	-	-	-
	FTT0842	-	Peptidoglycan-associated lipoprotein	9	17	-
	FTT0902	-	Protein of unknown function	8	1	-
	FTT0975	-	Protein of unknown function	-	-	-
	FTT1039	DacB1	d-Alanyl-d-alanine carboxypeptidase (penicillin-binding protein) family protein	-	-	-
	FTT1040	-	Lipoprotein	-	-	-
	FTT1334c	-	Hypothetical protein	1	1	-
	FTT1402c	-	Protein of unknown function	-	-	-
	FTT1538c	-	Hypothetical protein	25	22	-
	FTT1549	-	Protein of unknown function	-	-	-
	FTT1626c	-	Hypothetical protein	-	-	-
	FTT1688	-	Aromatic amino acid HAAP transporter	-	-	-
	FTT1314c	PilE6	Type IV pili fiber building block protein	4	-	-
	FTT1776c	-	Hypothetical protein	2	-	-
*3*	FTT0086	-	Conserved protein of unknown function	-	-	-
	FTT0103c	-	Hypothetical protein	2	-	-
	FTT0792	-	LPS locus	-	-	-
	FTT0889c	-	Type IV pili fiber building block protein	-	-	-
	FTT1530	FadB/AcbP	Bifunctional 3-hydroxacyl-CoA dehydrogenase	-	-	-
	FTT1564	-	Polyphosphate kinase	9	6	1
*4*	FTT0018	-	Secretion protein	10	-	-
	FTT0296	Pcp	Pyrrolidone-carboxylate peptidase	-	-	-
	FTT0482c	-	Lipoprotein	32	14	11
	FTT1029	DacD	d-Alanyl-d-alanine carboxypeptidase (penicillin binding protein) family protein	5	2	2
	FTT1157c	-	Type IV pili lipoprotein	-	-	-
	FTT1242	-	Hypothetical protein	-	-	-
	FTT1591	VacJ	Lipoprotein	-	-	-
	FTT1746	-	Peptidase	-	-	-
*5*	FTT1597	-	Putative uncharacterized protein	16	6	6
	FTT0522	-	Hypothetical protein, methyltransferase domain	-	-	-
	FTT0604	-	Hypothetical protein, methyltransferase domain	-	-	-
	FTT0677c	-	Putative uncharacterized protein	2	-	-

*Group 1*, multiple virulence association evidences and evidence about attenuation in an animal model; *group 2*, multiple virulence association evidences; *group 3*, transposon mutant screening evidence only; *group 4*, predicted secretion evidence only; *group 5*, orthology evidence only

### Y2H screening of host-pathogen interactions

**Fig. 1:**
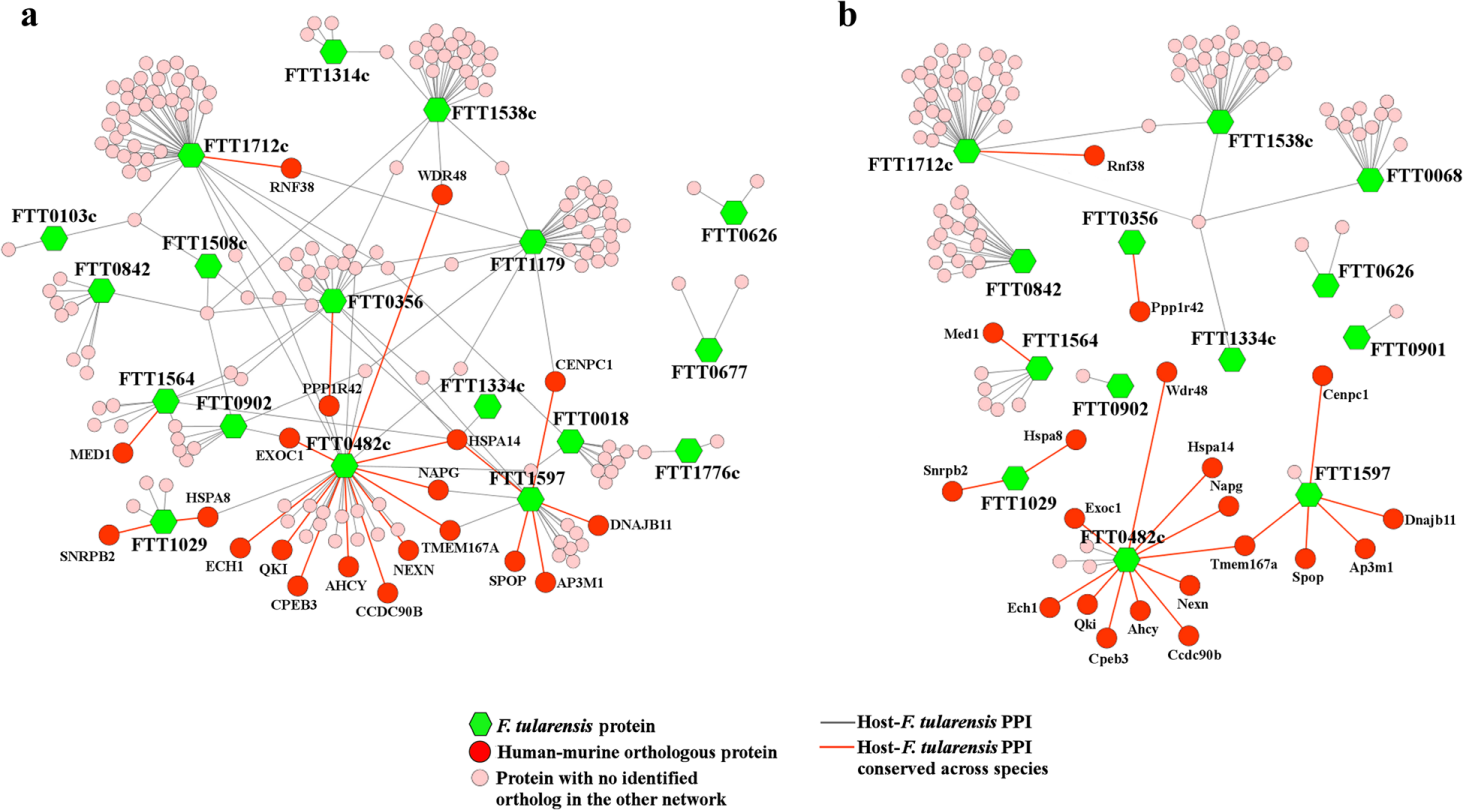
Yeast two-hybrid (Y2H) host-pathogen protein-protein interactions (PPIs). Using Y2H screens against whole human and murine proteome libraries, we identified 222 unique PPIs between 18 *Francisella tularensis* proteins and 183 human proteins (**a**) and 118 unique PPIs between 13 *F. tularensis* proteins and 113 murine proteins (**b**). Green nodes represent *F. tularensis* proteins, whereas pink and red nodes represent host proteins. Eleven *F. tularensis* proteins interacted with both hosts, and six of them participated in 21 conserved interactions (red edges), i.e., six *F. tularensis* proteins that interacted with both human proteins (red nodes in **a**) and their murine orthologs (red nodes in **b**)

All 49 putative and known virulence factors were successfully cloned, prepared, and tested in Y2H assays against both human and murine whole proteome libraries. Y2H screens identified 407 interactions between *F. tularensis* baits and human preys and 170 interactions between *F. tularensis* baits and murine preys. All baits were mapped to their corresponding *F. tularensis* locus tags, and all preys were mapped to their official gene symbols as defined in the HUGO Gene Nomenclature Committee database [50] or Mouse Genome Informatics database [51]. We were not able to map some of the identified preys to protein coding sequences, and interactions containing those preys were removed. Moreover, we removed protein interactions between *F. tularensis* and “sticky” host proteins known to be indiscriminate binders (F. Schwarz and M. Koegl, unpublished data). The final data consisted of 222 unique PPIs between 18 *F. tularensis* and 183 human proteins and 118 unique PPIs between 13 *F. tularensis* and 113 murine proteins (Fig. 1). Eleven *F. tularensis* proteins interacted with both hosts, seven interacted only with human proteins, and two interacted only with murine proteins. As shown in Fig. 1, the majority of *F. tularensis* proteins interacted with unique host proteins, i.e., 137 (75 %) human proteins and 110 (97 %) murine proteins interacted with a single *F. tularensis* protein. Furthermore, Y2H screens identified 21 conserved PPIs between these two data sets, i.e., interactions in which human proteins interacted with the same *F. tularensis* proteins as their murine orthologs. These conserved interactions consisted of six *F. tularensis* proteins and 20 unique host proteins. Table 2 shows the number of unique PPIs between each tested *F. tularensis* protein and human and murine proteins as well as the corresponding number of conserved interactions in terms of orthologous pairs of interactions. The small overlap between *F. tularensis*-human and *F. tularensis*-murine PPIs was potentially due to low quantities of a prey gene in one of the two cDNA libraries or to non-exhaustive sampling of host-prey and pathogen-bait protein interactions. However, the experimental conditions were the same for both sets of experiments. Thus, our detected interactions represent a subset of all interactions that can occur.

### Transposon mutant selection and in vitro growth

To identify a small set of *F. tularensis* protein candidates for validation in animal model experiments, we focused on nine proteins that interacted with multiple proteins in both hosts. From this set of proteins, we first removed two *F. tularensis* proteins that had already been tested for virulence attenuation in animal models: FTT0356 [44] and FTT0626 [43]. We then evaluated and ranked each of the remaining seven proteins based on the number of conserved interactions in which they participate or based on involvement of their host targets in biological processes and pathways related to virulence, e.g., signaling and apoptosis. This procedure resulted in seven previously uncharacterized virulence factor candidates: FTT0482c, FTT0842, FTT0902, FTT1029, FTT1538c, FTT1564, and FTT1597.

We successfully obtained from the transposon library insertion mutants in the *F. tularensis* subsp. *tularensis* Schu S4 strain for five virulence factor candidates (FTT0482c, FTT0902, FTT1538c, FTT1564, and FTT1597). We were unable to obtain an insertion mutant for the peptidoglycan-associated lipoprotein FTT0842 and FTT1029. Growth rate experiments in Chamberlain’s chemically defined growth medium [52] during the exponential phase showed that there was no significant difference in the growth rate for the mutants compared with those of the parent strain except for ΔFTT1564, which showed a minor growth enhancement (Additional file 3: Table S2).

The created transposon mutants are potentially subject to polar effects. Based on available computational bioinformatics information in the DOOR [53] and ProOpDB [54] databases, FTT0482c and FTT1597 belong to annotated single-gene operons; thus, they should not be associated with polar effects. On the other hand, FTT1538c belongs to an operon of four genes (FTT1537c to FTT1540c) and polar effects are theoretically possible. Further studies are required to definitively determine possible polar effects.

### Virulence attenuation in a murine intranasal infection model

**Fig. 2:**
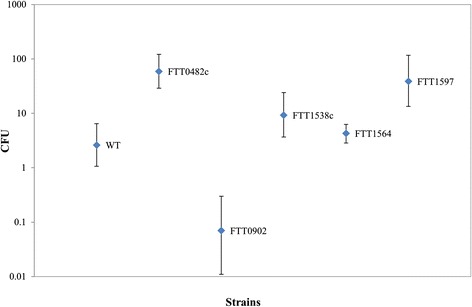
Fifty percent lethal dose (LD_50_) estimation. Using the dose-response experiments, we estimated LD_50_ values and 95 % confidence intervals for the wild-type strain and each of the mutant strains. The estimated wild-type LD_50_ of 2.62 CFU, where CFU denote colony-forming units, agreed with previously estimated values for the wild-type *F. tularensis* subsp. *tularensis* Schu S4 strain. The estimated LD_50_ values for ΔFTT0482c and ΔFTT1597 were higher than the wild-type value and outside its 95 % confidence intervals, whereas the LD_50_ value estimated for ΔFTT1538c was above the wild-type value, although with overlapping confidence intervals. These results support the notion that ΔFTT0482c, ΔFTT1538c, and ΔFTT1597 attenuate *F. tularensis* virulence in this animal model

Using intranasal infection, we gauged the effects of the five insertion mutations on *F. tularensis* virulence relative to the fully virulent wild-type *F. tularensis* subsp. *tularensis* Schu S4 strain. A total of 360 BALB/c mice [10 mice for each of six doses of increasing numbers of colony-forming units (CFUs) for each of the six strains (five mutant strains + the wild-type strain)] were exposed to different intranasal doses ranging from ≥0.03 CFU to ≥78,000 CFU and monitored for 21 days (Additional file 4: Table S3 and Additional file 5: Figure S1). We used these dose-response experiments to estimate the lethal dose for each strain and compare the potency of each strain. Estimated wild-type LD_50_ of 2.62 CFU (Fig. 2 and Table 3) agreed with previously estimated doses for the wild-type strain [9]. Table 3 also shows that the estimated LD_50_ values for ΔFTT0482c, ΔFTT1538c, and ΔFTT1597 were above the wild-type dose. Further analysis showed that the LD_50_ values for these three mutant strains were statistically larger (*p*-value ≤0.03) than the LD_50_ values of either the wild-type strain or the other two mutant strains (Additional file 6: Table S4).

**Table 3:** LD_50_ estimation

Strain	Log_10_(CFU) 95 % confidence intervals			CFU 95 % confidence intervals		
	Median	Lower	Upper	Median	Lower	Upper
Wild type	0.42	0.02	0.81	2.62	1.06	6.43
ΔFTT0482c	1.77^a^	1.46	2.08	59.14^a^	28.82	121.04
ΔFTT0902	-1.14	-2.01	-0.52	0.07	0.01	0.30
ΔFTT1538c	0.97^a^	0.57	1.38	9.26^a^	3.68	24.16
ΔFTT1564	0.63	0.46	0.80	4.29	2.86	6.26
ΔFTT1597	1.59^a^	1.13	2.07	38.66^a^	13.36	116.48

Estimated 50 % lethal dose (LD_50_) values for the *F. tularensis* subsp. *tularensis* Schu S4 wild-type strain and the following five mutant strains: ΔFTT0482c, ΔFTT0902, ΔFTT1538c, ΔFTT1564, and ΔFTT1597. CFU, colony-forming units. ^a^LD_50_ values above the wild-type LD_50_ value

The FTT0902 mutant showed a minute absolute increase in virulence that may be testing the limit of the experimental setup in terms of measureable CFUs. Although FTT0902 does not code for a known protein, it appears to have lipoprotein characteristics based on inspection of the amino terminus, potentially affecting the outer surface of *F. tularensis*. Such minor morphological changes may affect clumping of cells during the preparation of challenge doses and lead to an undercounting of the true dose. Conversely, the possibility that this mutant was really more virulent in the murine model of tularemia could not be ruled out based on our experiments, and, hence, we did not consider ΔFTT0902 for further evaluation.

**Fig. 3:**
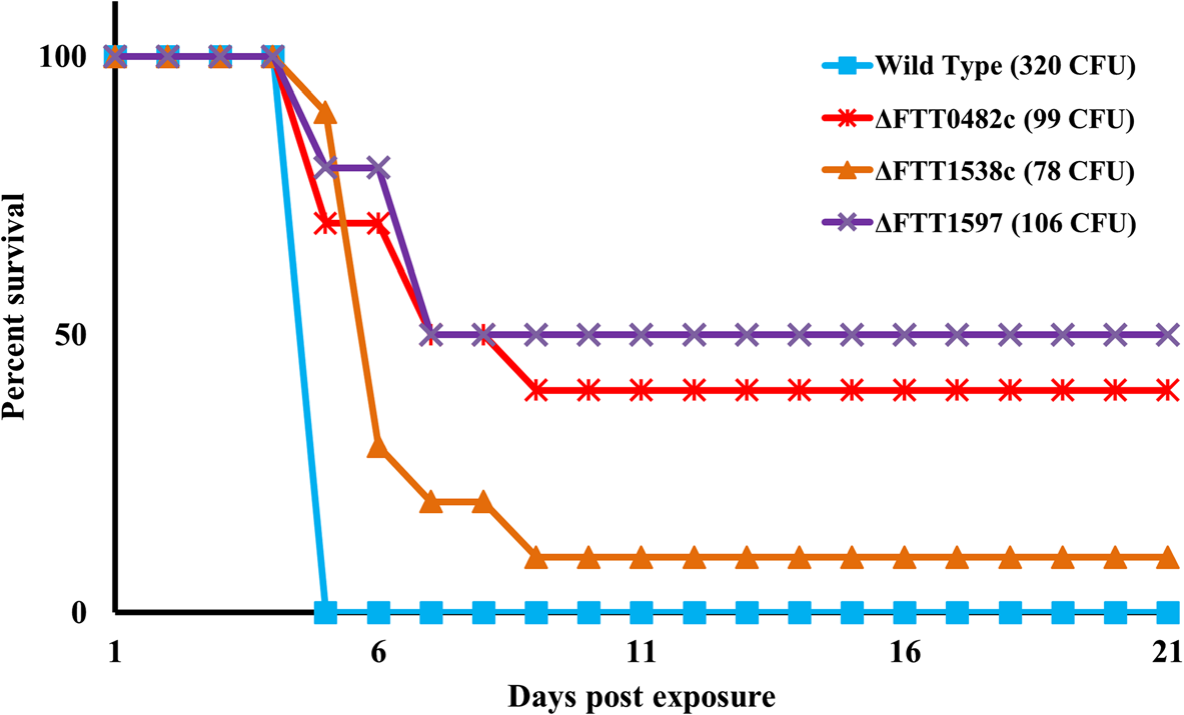
Mouse intranasal challenge model results. We compared the survival rates of 30 (3 × 10) mice exposed to the ΔFTT0482c, ΔFTT1538c, and ΔFTT1597 mutants for intranasal doses of ≥78 CFUs, with 10 mice exposed to wild-type strain doses of 320 CFU. Animals were monitored for 21 days. All mice exposed to the high-dose of the wild-type strain died by the end of the fifth day (blue line), whereas mice exposed to mutant strains had a slower time to death and survived in larger numbers 21 days post-exposure (purple, red, and orange lines). There was a statistically significant difference in the survival rate of mice exposed to mutant strains and mice exposed to the wild-type strain (*p*-value ≤10^-3^). These results support the notion that each of the three mutants attenuates virulence when infected in mice via the intranasal route of infection

Given these results, we compared the survival curves of mice exposed to the ΔFTT0482c, ΔFTT1538c, and ΔFTT1597 mutant strains for intranasal doses of ≥78 CFU (≥30 LD_50_) to mice exposed to a wild-type strain dose of 320 CFU (122 LD_50_). Figure 3 shows that on the fifth day, all wild-type mice exposed to the high-dose died. Conversely, mice exposed to mutant strains tended to have a slower time to death (nine of ten mice exposed to ΔFTT1538c died by *day 9*) as well as survived in larger numbers (five mice exposed to ΔFTT1597 and four mice exposed to ΔFTT0482c survived 21 days post-exposure). A survival-curve analysis indicated that the difference in survival times between mutant strains and the wild-type strain was statistically significant (*p*-value ≤10^-3^). Although the values in terms of CFUs are modest for the three mutants, and may not be of biological significance for FTT1538c, all results showed a consistent increase in CFUs. Thus, these results demonstrate that these three mutants showed attenuated virulence when used to challenge BALB/c mice via the intranasal route of infection and, therefore, implicated three *F. tularensis* proteins not previously considered factors for in vivo virulence.

### Pairwise Y2H experiments for select human-*F. tularensis* virulence factors PPIs

To further assess confidence in the identified host-pathogen interactions from our high-throughput Y2H screen, we retested a small number of host-*F. tularensis* protein interactions using pairwise Y2H assays. We focused only on interactions including the identified novel virulence factors (FTT0482c, FTT1538c, and FTT1597) and their interacting human protein partners. To select the human protein interaction partners from the larger data set, we examined and ranked each protein based on involvement in biological processes or pathways associated with *F. tularensis* pathogenicity, conservation among species, and ability to interact with more than one of the three virulence factors. Using this procedure, we selected 12 human-*F. tularensis* interactions for retesting and confirmed four interactions in this assay: FTT0482c with WD repeat-containing protein 48 (WDR48), FTT1538c with 78-kDa glucose-regulated protein (HSPA5), FTT1538c with WDR48, and FTT1597 with AP-3 complex subunit mu-1 (AP3M1). The success rate of 33 % was less than the expected 50-80 % rate, although not necessarily out of line with earlier reports of retesting Y2H interactions using similar assay conditions [55–57]. One reason for the lower reproducibility rate was that the isoforms of the genes used for the retests might not be identical to what we identified in the high-throughput cDNA library screening, as the sequencing reads for the preys provide information for only part of the cDNA. In the retesting, we used the additionally constructed human clones to identify three new interactions that were not detected in our original high-throughput screening: FTT1538c/AP3M1, FTT1597/WDR48, and FTT1597/Quaking (QKI). All retest results are shown in supplemental information (Additional file 7: Table S5).

The pairwise retesting of the Y2H interactions does not constitute a methodology-independent verification of the initial screening results. We attempted two independent approaches to verify select interaction from the Y2H assays, i.e., *1*) one bait-one prey interaction verification by recombinant expression of bait and prey genes followed by co-immunoprecipitation and LUminescence-based Mammalian IntERactome (LUMIER) assays and *2*) affinity purification/mass spectrometry (AP/MS) assays. We restricted these studies to three *F. tularensis* targets – FTT1597, FTT0482c, and FTT1564 – that interacted with four human proteins. Although we successfully cloned all three selected *F. tularensis* genes and three of the four selected human genes into the LUMIER expression vectors and transfected them into the human HEK293T cell line, we were essentially unable to express the bacterial proteins using multiple expression vectors. It may not be biologically feasible to express the selected bacterial genes in mammalian cell lines, as complex folding and post-translational processing may not be reproducible in the in vitro environment.

## Discussion

### Using Y2H methods to detect *Francisella*-human protein interactions

Protein-interaction detection based on random cDNA libraries and Y2H technology is associated with well-known experimental and biological biases leading to mixed confidence and confusion about the reliability of the observed interactions. Prime is the often-noted non-repeatability of observed Y2H interactions, primarily stemming from insufficient retesting [58]. It is not practical to repeat screens exhaustively to recover all possible Y2H interaction high-throughput experiments. Although more testing increases confidence of multiple observed interactions, the absence of an interaction may not indicate that the interaction cannot occur, just that the experimental or biological conditions were not amenable to sample that interaction. A further uncertainty on the biological level is that presence of an Y2H interaction does not necessarily indicate that the interaction actually occurs, just that it can occur.

*Comparison of high*-*throughput data sets*. Our study is not the first study to screen for interactions between human and *F. tularensis* proteins. However, it is the first study to systematically use targeted screens to identify potential *F. tularensis* virulence factors and PPIs associated with secretion system proteins. In contrast, Dyer et al. [59] used a random genome library of *F. tularensis* as baits (DNA-binding domain) and human cDNA libraries as preys (activation domain) to identify host-pathogen interactions using Y2H technology. This study reported 1,383 host-pathogen interactions between 999 human and 415 *F. tularensis* proteins. We found that 24 proteins from our list of 49 *F. tularensis* putative and known virulence factors were present in the Dyer et al. data set, with nine proteins associated with 180 interactions in our data set. Although these nine proteins participated in 43 PPIs in the Dyer et al. study, there was no overlap among these interactions and our data set. This result is again a consequence of the variations in the libraries, Y2H vectors, and strains used in terms of different bait/prey clone properties and laboratory procedures. For example, Dyer et al. screened random fragments of *F. tularensis* genomic DNA, whereas we used full-length ORF clones. Similar low overlaps have been noted among *Helicobacter pylori* proteins [56] and for *Yersinia pestis* host-pathogen PPIs obtained by random library and systematic Y2H screening [60].

### Analysis of virulence factor interactions and infectivity mechanisms

We have chosen to organize the discussion of virulence factor interactions with host proteins into three sections (I-III) based on the level of interaction confidence. Thus, the first analysis was based on highest confidence data, consisting of the successfully retested Y2H interactions, for the three novel in vivo validated virulence factors identified here. Second, we used all Y2H data for the two previously known virulence factors included in our data set to assess their possible contribution to *Francisella* pathogenicity. Finally, we present an analysis of potential *Francisella* infectivity mechanisms that factors in all detected Y2H interactions in identifying targeted human proteins and protein interaction networks.

### I – Novel virulence factors and their PPI-inferred virulence phenotypes

Little prior knowledge exists on the function or role of virulence factor proteins FTT0482c, FTT1538c, and FTT1597; only FTT0482c has been assigned functionality as a hypothetical lipoprotein. Here, we positively identified them as virulence factors that do not influence normal bacterial growth yet affect lethality in an intranasal murine challenge model. Furthermore, we can hypothesize on their possible roles using the measured Y2H interaction profiles with human proteins in promoting *Francisella* intracellular lifestyle and pathogenicity.

The interactions of FTT1538c with AP3M1, WDR48, and HSPA5 strongly link this protein to biological processes involved in human intracellular transport. AP3M1 is a component of an adaptor complex involved in the Golgi to endosome/lysosome intracellular protein trafficking, in particular from the early endosome to the late endosome compartment [61]. Binding to and interfering with this adaptor complex could alter endosome maturation or endosome-lysosome fusion. Similarly targeting lysosomal degradation processes, FTT1538c binding or interacting with WDR48, known to regulate deubiquitinating complexes [62], could provide a mechanism to interfere with ubiquitin tagging of pathogen protein and prevent their targeted destruction. HSPA5 belongs to the family of 70-kDa heat shock proteins generally found in the endoplasmic reticulum; however, it is associated with multiple cellular locations and functions, including the unfolded protein response, protein transport, and interactions with apoptotic executors [63].

Consistent with these interactions/targets, host targets identified in the high-throughput Y2H include SNARE protein STX8, a protein known to be required for late endosome trafficking [63], and PI4K2B, involved in overall phosphatidylinositol 4-kinase activity and protein trafficking [64]. Furthermore, GO enrichment analysis of all host proteins that interact with FTT1538c also indicated enrichment of membrane-bound vesicular proteins (p_adj_ = 0.05). Thus, the interaction data are compatible with FTT1538c contributing to intracellular survival by interfering with vesicular trafficking, possibly during endosome and lysosome fusion during phagocytosis, and ultimately contributing to phagosomal escape.

Although lacking functional annotation, FTT1597 contains tetratricopeptide repeat motifs recently shown to be present in other proteins implicated in *F. tularensis* virulence [65]. The similarity in interactions with AP3M1 and WDR48 highlight a possible functional similarity to FTT1538c. Further linking the functionalities, in the high-throughput Y2H data set, FTT1597 interacted with DNAJB11, a co-chaperone of HSPA5. On the other hand, the observed interaction with QK1, an RNA-binding protein that regulates mRNA activities, is not known to be linked to any of these functionalities. Lipoprotein FTT0482c interacted with WDR48, which thus was a common host interaction among all the identified virulence factors. Five other interacting host proteins in high-throughput screening share this involvement in targeting ubiquitination related proteins/processes (ANAPC10, CACYBP, RANBP2, UBE2V2, and XPA). In these experiments, FTT0482c also interacted with the renin receptor ATP6AP2, which activates the ERK inflammatory pathway and whose inhibition is associated with decreased phagocytosis [66].

### II – Phenotypic associations of known virulence factors

Turning to the interaction analysis of known virulence factors based on the high-throughput Y2H screens, we examined identified PPIs for known virulence factors and those highly implicated in virulence for binding to host protein targets. A lack of host protein interactions does not necessarily imply a lack of a biological role in virulence, as virulence factor proteins may be involved in functions other than protein binding as detectable by the Y2H screens.

Our screens identified a total of 76 and 12 interactions for FTT1712c (IglC2) and FTT0068 (SodB), respectively (Table 2). IglC2 interacted with proteins from both hosts, whereas SodB interacted only with murine proteins. IglC2 is a *Francisella* pathogenicity island protein (and a member of T6SS), which has already been identified as a protein required for intracellular growth, phagosomal escape, regulation of host signaling, and apoptosis [7, 67–69]. We identified 43 interactions between IglC2 and human proteins and 33 interactions between IglC2 and murine proteins, the largest number of interactions detected for any *F. tularensis* protein in our screens. We found that IglC2 interacted with a set of proteins that are linked to the inflammatory response and activation of cAMP signaling pathway stimulation via mitogen-activated protein kinases, i.e., phosphatidylinositol 3,4,5-trisphosphate 3-phosphatase (PTEN), fibroblast growth factor receptor substrate 2 (FRS2), and transcription factor AP-1 (JUN). As these proteins are intimately linked to host cell defense and immune responses [10, 70], they provide important targets for immune suppression and the successful establishment of an infection.

For SodB that has been identified as a factor in *F. tularensis* oxidative stress protection [71], we found multiple potential interacting partners, including interactions with the murine thioredoxin-interacting protein Txnip, a protein mediating oxidative stress via thioredoxin activity [72].

Furthermore, we identified interactions for five additional proteins from the first group of proteins, i.e., those highly implicated in virulence (Table 2). For two of those, heat shock protein FTT0356 (HtpG) [11] and GTP-binding translational elongation factor FTT1179 (BipA) [43], we found interactions with human proteins but only one murine interaction, even though murine orthologs exists. For the remaining three proteins (LpnA, RelA, and Lon) we only found a scatter of interactions that did not point toward any specific protein interaction pattern or biological process.

### III – Characterization of *F. tularensis* infectivity based on its host targets

It was not possible to perform a statistically meaningful evaluation of the roles of each individual *F. tularensis* protein due to the overall small number of identified host-pathogen protein interactions. Instead, given that the selected set of 49 bacterial proteins was strongly biased toward secreted/virulence associated protein, we used the aggregated host-pathogen interaction data set to investigate host molecular mechanisms targeted by the selected subset of *F. tularensis* proteins. The aggregated data set contains all experimentally detected human-*F. tularensis* PPIs and computationally derived human (murine orthologs)-*F. tularensis* PPIs and consists of 298 unique interactions between 18 *F. tularensis* and 249 human proteins.

First, we performed enrichment analyses based on GO annotation as outlined in the METHODS. Table 4 shows that *F. tularensis* proteins target a statistically significant number of host proteins located in or around the endoplasmic reticulum and Golgi compartments. Known pathogen targets in these compartments are linked to activation of protein degradation processes [73].

**Table 4:** Enrichment of GO terms for human proteins interacting with *F. tularensis*

	GO term ID	GO term description	Number of proteins	*p*-value	FDR
Cellular localization	GO:0005793	Endoplasmic reticulum-Golgi intermediate compartment	4	2.7∙10^-3^	0.04
	GO:0005788	Endoplasmic reticulum lumen	6	1.7∙10^-3^	0.03
	GO:0005801	*Cis*-Golgi network	3	3.6∙10^-3^	0.05
	GO:0005720	Nuclear heterochromatin	3	3.6∙10^-3^	0.05
	GO:0042470	Melanosome	7	9.5∙10^-5^	0.00
	GO:0042588	Zymogen granule	3	3.3∙10^-3^	0.01
	GO:0035097	Histone methyltransferase complex	4	3.8∙10^-3^	0.05
	GO:0031519	PcG protein complex	3	5.1∙10^-3^	0.06
Molecular function	GO:0051082	Unfolded protein binding	6	1.9∙10^-3^	0.18
	GO:0019904	Protein domain specific binding	14	3.6∙10^-3^	0.18

*FDR,* false discovery rate calculated using Benjamini and Hochberg multiple test correction [40]; *GO,* Gene Ontology

Second, we used the host PPI network to investigate the influence of the pathogen on host proteins and interactions between host proteins [20]. We mapped proteins from the merged human-*F. tularensis* PPI data set onto a human PPI network consisting of 76,043 physical PPIs among 11,688 proteins [41]. Of the 249 human proteins interacting with *F. tularensis* proteins, 194 (~78 %) were found in the human network. Furthermore, we found that 52 of these human proteins were a part of the LCC, i.e., the largest subnetwork in which a path connects every two proteins to each other. The size of this LCC was significantly larger than what could be expected by random chance in the human PPI network, i.e., *N*_ran_ = 9.1, σ_ran_ = 6.4. Within this LCC, we identified 139 sets of human PPIs in which, in each set, all human proteins interacted with at least one of the 18 *F. tularensis* proteins and had the same GO biological process annotations; we denoted these sets as interaction modules [20]. Table 5 shows 12 interaction modules that correspond to the most specific (lowest level) GO annotations of all identified interaction modules. These interaction modules are associated with biological processes related to cellular regulation, signaling, cellular response, and protein modification.

**Table 5:** Enrichment of GO biological processes in host subnetworks

Category	Term		Size		*p*-value		
	ID	Description	*N*_C_	*N*_M_	p_GO_	p_Rp_	p_Rn_
Cellular regulation	GO:0045944	Positive regulation of transcription from RNA polymerase II promoter	7	4	6.4∙10^-3^	1.0∙10^-4^	1.5∙10^-4^
	GO:0045892	Negative regulation of transcription, DNA-dependent	11	6	0.0	0.0	1.3∙10^-4^
	GO:0051090	Regulation of sequence-specific DNA binding transcription factor activity	6	4	1.1∙10^-3^	0.0	1.1∙10^-4^
	GO:0051345	Positive regulation of hydrolase activity	8	5	4.0∙10^-4^	0.0	7.7∙10^-5^
	GO:0051726	Regulation of cell cycle	9	4	6.0∙10^-4^	7.0∙10^-4^	1.1∙10^-3^
Cellular signaling	GO:0023052	Signaling	22	8	9.2∙10^-3^	0.0	1.1∙10^-3^
	GO:0007166	Cell surface receptor signaling pathway	15	5	3.4∙10^-3^	6.0∙10^-4^	1.8∙10^-3^
Cellular response	GO:0070887	Cellular response to chemical stimulus	17	7	0.0	0.0	4.0∙10^-4^
	GO:0006950	Response to stress	18	10	4.0∙10^-3^	0.0	4.5∙10^-4^
	GO:0010033	Response to organic substance	17	8	0.0	0.0	4.6∙10^-4^
Protein modification	GO:0016567	Protein ubiquitination	9	4	0.0	1.0∙10^-4^	2.9∙10^-5^
	GO:0016310	Phosphorylation	10	5	9.7∙10^-3^	2.0∙10^-4^	1.1∙10^-4^

*N*_C_, number of proteins in the largest connected component annotated with a given term; *N*_M_, number of proteins in the largest interaction module for a given term; p_GO_, probability of the same number of proteins as *N*_C_ being annotated with a given GO term solely through a random selection; p_Rn_, probability that a given number of proteins as *N*_M_ are annotated with a given GO term solely through random selection in a random network that has the same degree distribution as the human network; p_Rp_, probability that a given number of proteins as *N*_M_ are annotated with a given GO term solely through random selection. This table contains only the largest statistically significant interaction module for each term; the complete list is available as supplementary information (Additional file 8: Table S6).

**Fig. 4:**
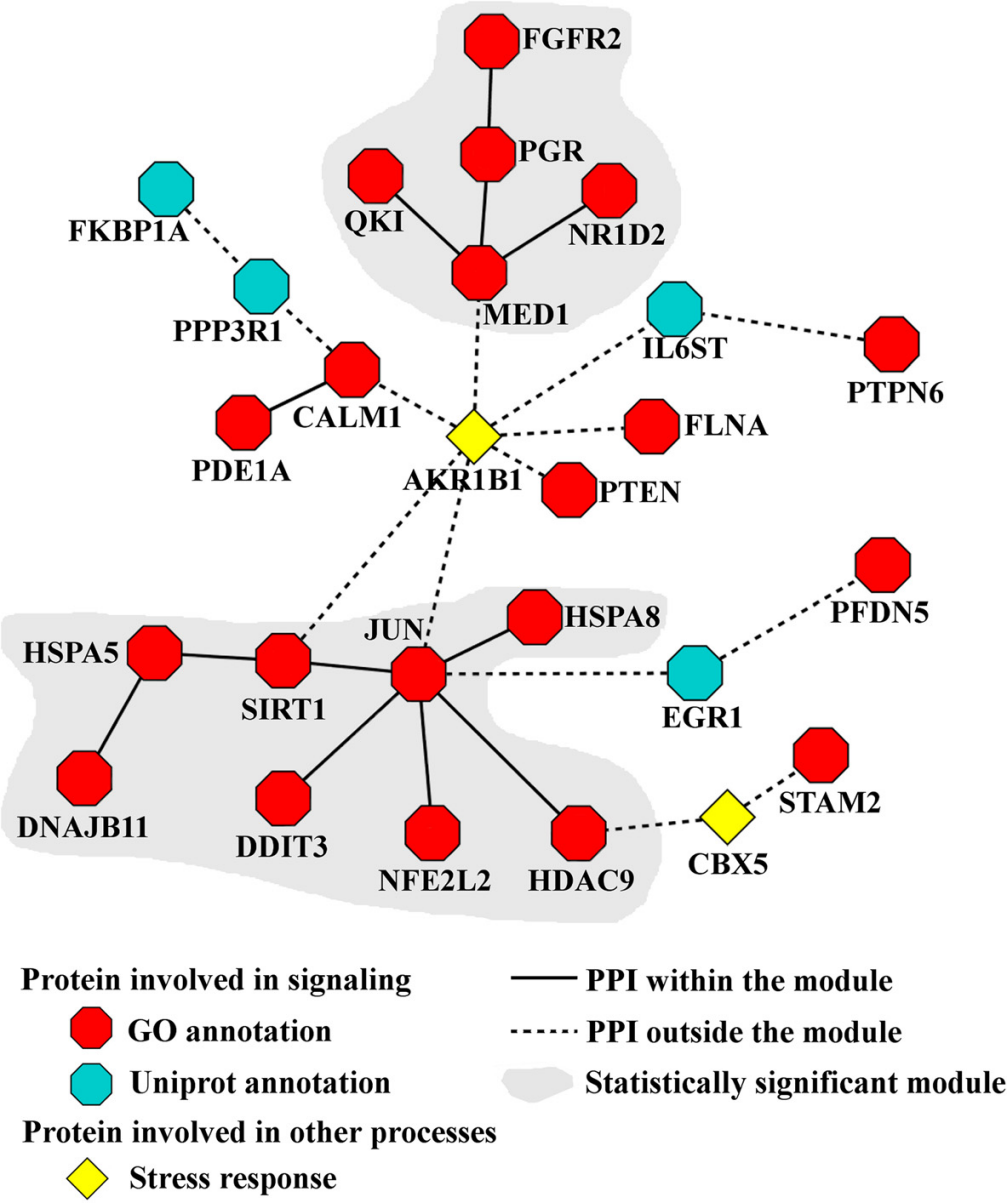
Host signaling and the stress response as a *F. tularensis* virulence factor target. *F. tularensis* proteins target a large number of host proteins involved in signaling (red and blue octagons) and the stress response (yellow diamond). Using the connectivity and Gene Ontology (GO) annotation, we identified two statistically significant signaling interaction modules, one containing eight host proteins and one containing five host proteins (shaded area). The larger one contains proteins related to the immune system response, whereas the smaller one contains proteins related to intracellular signaling. Further annotation assessment of proteins from the largest connected component (LCC) revealed additional proteins associated with signaling (blue octagons) and existing interaction modules. These results suggest that *F. tularensis* targets host proteins involved in signaling to interfere with cell-to-cell signaling and the immune response but also points toward PPIs among these host proteins as equally important targets

It has been shown that a number of bacterial pathogens interfere with host signaling pathways to obstruct host defense systems and promote pathogen colonization [74–76]. Indeed, the largest number of proteins in the LCC was involved in signaling, and we identified two statistically significant signaling interaction modules, containing eight and five host proteins (Fig. 4, shaded areas). The larger interaction module contained proteins related to defense responses, whereas the smaller interaction module mostly contained proteins related to intracellular signaling. Our analysis also identified 18 stress response proteins in the LCC, 16 of which were also annotated as signaling proteins. One of the remaining two proteins was aldo-keto reductase family 1 protein (AKR1B1), a protein involved in the stress response [77], which links the two interaction modules. The other stress response protein, chromobox protein homolog 5 (CBX5), links the larger signaling interaction module and signal transducing adapter molecule 2 (STAM2), which is associated with the immune system response. Further assessment of proteins from the LCC revealed additional proteins associated with signaling (blue octagons) [78]. Overall, the identified signaling stress response component consists of 50 % of proteins from the LCC (26 proteins). These results suggest that proteins involved in cell-to-cell signaling and innate immune defense responses are potential targets for *Francisella*; furthermore, it also points toward the interactions among these host proteins as equally important targets.

## Conclusions

Direct interactions with specific host proteins and the ability to influence host PPIs are important components for intracellular pathogens to avoid host-cell defense mechanisms and successfully establish an infection. Although direct host-pathogen protein-protein binding is only one aspect of virulence, it is a critical component in directly manipulating and interfering with cellular processes in the host cell. Here, we exploited this using an integrated bioinformatics/proteomics method to identify both virulence factors and their complement of interacting host proteins.

The implemented approach of selecting a focused set of putative virulence factors based on multiple evidence, down-selection through in vitro high-throughput Y2H screening, and final assessment using mutants in an animal infection model was highly effective. Of the final five mutants selected for animal testing, three showed statistically significant virulence attenuation that was not directly attributable to gross bacterial growth defects of the tested mutants. Furthermore, the up-front Y2H screening and subsequent retesting of select host-pathogen protein interactions provided indications as to possible virulence mechanisms associated with the direct binding of the virulence factors to host proteins. This led to an initial hypothesis as to which host processes were targeted by the three virulence factors FTT0482c, FTT1538c, and FTT1597, in particular identifying intracellular protein trafficking between the early endosome to the late endosome as an important component in virulence attenuation. We also used the Y2H data to investigate host protein binding of two known virulence factors, showing that direct protein binding was a component in the modulation of the inflammatory response via activation of mitogen-activated protein kinases and in the oxidative stress response.

### Availability of supporting data

The data sets supporting the results of this article are included within the article and its additional files.

## Additional files

Additional file 1: Table S1.
*Francisella* strains used to identify proteins overrepresented in the pathogenic strains. (DOCX 27 kb) Additional file 2: PPI data.
*F. tularensis* protein interactions with *1*) human proteins, *2*) murine proteins, and *3*) the combined set of human proteins augmented with murine orthologs. (XLSX 28 kb) Additional file 3: Table S2. Exponential-phase growth rates. (DOCX 20 kb) Additional file 4: Table S3. Mouse intranasal infection results. (DOCX 28 kb) Additional file 5: Figure S1. Evaluation of the effect of five mutants on *Francisella tularensis* virulence using mouse intranasal model experiments. (DOCX 372 kb) Additional file 6: Table S4. Likelihood that one strain is more or less potent than any other strain. (DOCX 18 kb) Additional file 7: Table S5. Human-*F. tularensis* protein-protein interactions retested in pairwise Y2H experiments. (DOCX 20 kb) Additional file 8: Table S6. Enrichment of GO biological processes in host subnetworks. (DOCX 63 kb)

## Abbreviations

3-AT: 3-amino-1,2,4-triazole
AKR1B1: Aldo-keto reductase family 1 protein
AP3M1: AP-3 complex subunit mu-1
CBX5: Chromobox protein homolog 5
CFU: Colony-forming unit
ETA: Enhanced tularemia agar
ETB: Enhanced tularemia broth
FRS2: Fibroblast growth factor receptor substrate 2
GO: Gene Ontology
HSPA5: 78-kDa glucose-regulated protein
IBC: Institutional Biosafety Committee
JUN: Transcription factor AP-1
LCC: Largest connected component
LD_50_: 50 % lethal dose
NCBI: National Center for Biotechnology Information
OD_600_: Optical density measurements at 600 nm
ORF: Open reading frame
PPI: Protein-protein interaction
PTEN: Phosphatidylinositol 3,4,5-trisphosphate 3-phosphatase
QKI: Quaking
STAM2: Signal transducing adapter molecule 2
T2SS: Type II secretion system
T6SS: Type VI secretion system
USAMRIID: United States Army Medical Research Institute for Infectious Diseases
WDR48: WD repeat-containing protein 48
Y2H: Yeast two-hybrid
YEPDA: Yeast extract, peptone, dextrose, and adenine
