# Supplementary material for: Using host-pathogen protein interactions to identify and characterize Francisella tularensis virulence factors
Source: BMC Genomics. 2015 Dec 29;16:1106. doi: 10.1186/s12864-015-2351-1 (PMC4696196; doi:10.1186/s12864-015-2351-1)
Supplement: Additional file 1: Table S1. — Francisella strains used to identify proteins overrepresented in the pathogenic strains. (DOCX 27 kb) [file 12864_2015_2351_MOESM1_ESM.docx]

**Table S1.** *Francisella strains used to identify proteins overrepresented in the pathogenic strains*

|  | **Genome** | **Size, Mb** | **PATRIC Coding Sequences^a^** | **RefSeq Coding Sequences^b^** | **Status** |
| --- | --- | --- | --- | --- | --- |
| Highly pathogenic  *F. tularensis* genomes | *F. tularensis* subsp *tularensis* Schu S4 | 1.89 | 2,097 | 1,603 | Complete |
|  | *F. tularensis* subsp *tularensis* FSC033 | 1.85 | 2,090 | 1,523 | WGS |
|  | *F. tularensis* subsp *tularensis* FSC198 | 1.89 | 2,095 | 1,605 | Complete |
|  | *F. tularensis* subsp *tularensis* MA00-2987 | 1.87 | 2,148 | 1,640 | WGS |
|  | *F. tularensis* subsp *tularensis* NE061598 | 1.89 | 2,111 | 1,836 | Complete |
|  | *F. tularensis* subsp *tularensis* WY96-3418 | 1.90 | 2,082 | 1,634 | Complete |
| Less pathogenic *F. tularensis* genomes | *F. tularensis* subsp *mediasiatica* FSC147 | 1.89 | 2,170 | 1,406 | Complete |
|  | *F. tularensis* subsp *holarctica* 257 | 1.89 | 2,312 | 1,501 | WGS |
|  | *F. tularensis* subsp *holarctica* | 1.90 | 2,132 | 1,754 | Complete |
|  | *F. tularensis* subsp *holarctica* FSC022 | 1.87 | 2,121 | 1,521 | WGS |
|  | *F. tularensis* subsp *holarctica* FSC200 | 1.79 | 2,057 | 1,993 | WGS |
|  | *F. tularensis* subsp *holarctica* FTNF002-00 | 1.89 | 2,163 | 1,580 | Complete |
|  | *F. tularensis* subsp *holarctica* OSU18 | 1.90 | 2,177 | 1,555 | Complete |
|  | *F. tularensis* subsp *holarctica* URFT1 | 1.80 | 2,036 | N/A | WGS |
| Less pathogenic *F. novicida* genomes | *F.* cf *novicida* Fx1 | 1.91 | 1,808 | 1,818 | Complete |
|  | *F. novicida* FTE | 1.89 | 1,806 | 1,772 | WGS |
|  | *F. novicida* FTG | 1.87 | 1,766 | 1,734 | WGS |
|  | *F. novicida* GA99-3548 | 1.89 | 1,798 | 1,659 | WGS |
|  | *F. novicida* GA99-3549 | 1.90 | 1,835 | 1,664 | WGS |
|  | *F. novicida* U112 | 1.91 | 1,821 | 1,719 | Complete |
|  | *F.* cf *novicida* 3523 | 1.95 | 1,863 | 1,854 | Complete |

^a^The number of coding sequences for each of the genomes that are available in the PathoSystems Resource Integration Center (PATRIC) database [[1](#_ENREF_1)]. ^b^The number of coding sequences available in the National Center for Biotechnology Information Reference Sequence database [[2](#_ENREF_2)]. WGS, whole genome sequencing.

References

1. Gillespie JJ, Wattam AR, Cammer SA, Gabbard JL, Shukla MP, Dalay O et al. PATRIC: The comprehensive bacterial bioinformatics resource with a focus on human pathogenic species. Infect Immun. 2011;79(11):4286-98.

2. Pruitt KD, Tatusova T, Brown GR, Maglott DR. NCBI Reference Sequences (RefSeq): Current status, new features and genome annotation policy. Nucleic Acids Res. 2012;40(Database issue):D130-D5.
