# Supplementary material for: Using host-pathogen protein interactions to identify and characterize Francisella tularensis virulence factors
Source: BMC Genomics. 2015 Dec 29;16:1106. doi: 10.1186/s12864-015-2351-1 (PMC4696196; doi:10.1186/s12864-015-2351-1)
Supplement: Additional file 3: Table S2. — Exponential-phase growth rates. (DOCX 20 kb) [file 12864_2015_2351_MOESM3_ESM.docx]

**Table S2.** *Exponential-phase* *growth rates*

| **Strain** | **Mean Doubling Time, min** | | | **Standard Deviation, min** | | |
| --- | --- | --- | --- | --- | --- | --- |
|  | *Experiment 1* | *Experiment 2* | *Experiment 3* | *Experiment 1* | *Experiment 2* | *Experiment 3* |
| Wild type | 94.3 | 96.0 | 95.2 | 5.5 | 3.3 | 2.7 |
| ΔFTT0482c | 94.9 | 104.9 | 105.5 | 4.3 | 4.5 | 2.8 |
| ΔFTT0902 | 90.5 | 96.9 | 111.6 | 5.0 | 3.5 | 5.8 |
| ΔFTT1597 | 96.0 | 100.0 | 99.2 | 15.5 | 4.7 | 4.1 |
| ΔFTT1538c | 93.9 | 95.5 | 95.6 | 4.2 | 3.3 | 3.2 |
| ΔFTT1564* | 86.2 | 88.9 | 90.7 | 3.8 | 3.1 | 3.2 |
| ΔFTT1597 | 96.0 | 100.0 | 99.2 | 15.5 | 4.7 | 4.1 |

Triplicate measurements of the exponential-phase growth rates [[1](#_ENREF_1)] for *F. tularensis* Schu S4 wild-type strain and five mutant strains: ΔFTT0482c, ΔFTT0902, ΔFTT1538c, ΔFTT1564, and ΔFTT1597. *Growth rate different from wild-type value (p-value = 0.03).

References

1. Hall BG, Acar H, Nandipati A, Barlow M. Growth rates made easy. Mol Biol Evol. 2014;31(1):232-8.
