# Supplementary material for: Using host-pathogen protein interactions to identify and characterize Francisella tularensis virulence factors
Source: BMC Genomics. 2015 Dec 29;16:1106. doi: 10.1186/s12864-015-2351-1 (PMC4696196; doi:10.1186/s12864-015-2351-1)
Supplement: Additional file 4: Table S3. — Mouse intranasal infection results. (DOCX 28 kb) [file 12864_2015_2351_MOESM4_ESM.docx]

**Table S3.** *Mouse intranasal infection results*

| **Strain** | **Dose, CFU** | **Log(Dose)** | **Alive** | **Dead** | **Total** | **Proportion Dead** | **Time to Death, day** | |
| --- | --- | --- | --- | --- | --- | --- | --- | --- |
|  |  |  |  |  |  |  | **Mean** | **SD** |
| Wild type | 3200.0 | 3.51 | 0 | 10 | 10 | 1.0 | 4.60 | 0.52 |
|  | 320.0 | 2.51 | 0 | 10 | 10 | 1.0 | 5.00 | 0.00 |
|  | 32.0 | 1.51 | 1 | 9 | 10 | 0.9 | 5.67 | 0.50 |
|  | 3.2 | 0.51 | 4 | 6 | 10 | 0.6 | 6.00 | 0.00 |
|  | 0.3 | -0.49 | 9 | 1 | 10 | 0.1 | 6.00 | - |
|  | 0.03 | -1.49 | 10 | 0 | 10 | 0.0 | - | - |
| ΔFTT0482c | 9900.0 | 4.00 | 0 | 10 | 10 | 1.0 | 4.50 | 0.53 |
|  | 990.0 | 3.00 | 0 | 10 | 10 | 1.0 | 4.90 | 0.32 |
|  | 99.0 | 2.00 | 4 | 6 | 10 | 0.6 | 6.33 | 1.63 |
|  | 18.14 | 1.26 | 8 | 2 | 10 | 0.2 | 6.00 | 1.41 |
|  | 1.6 | 0.20 | 10 | 0 | 10 | 0.0 | - | - |
|  | 0.14 | -0.85 | 10 | 0 | 10 | 0.0 | - | - |
| ΔFTT0902 | 32000.0 | 4.51 | 0 | 10 | 10 | 1.0 | 4.00 | 0.00 |
|  | 3200.0 | 3.51 | 0 | 10 | 10 | 1.0 | 4.40 | 0.52 |
|  | 320.0 | 2.51 | 0 | 10 | 10 | 1.0 | 4.40 | 0.84 |
|  | 3.2 | 0.51 | 0 | 10 | 10 | 1.0 | 5.20 | 0.42 |
|  | 0.3 | -0.49 | 5 | 5 | 10 | 0.5 | 6.00 | 0.71 |
|  | 0.03 | -1.49 | 5 | 5 | 10 | 0.5 | 6.20 | 0.84 |
| ΔFTT1538c | 78000.0 | 4.89 | 0 | 10 | 10 | 1.0 | 4.40 | 0.52 |
|  | 7800.0 | 3.89 | 0 | 10 | 10 | 1.0 | 4.80 | 0.42 |
|  | 780.0 | 2.89 | 0 | 10 | 10 | 1.0 | 5.10 | 0.32 |
|  | 78.0 | 1.89 | 1 | 9 | 10 | 0.9 | 6.33 | 1.12 |
|  | 7.8 | 0.89 | 6 | 4 | 10 | 0.4 | 6.75 | 0.23 |
|  | 0.8 | -0.11 | 9 | 1 | 10 | 0.1 | 6.00 | - |
| ΔFTT1564 | 43000.0 | 4.63 | 0 | 10 | 10 | 1.0 | 4.90 | 0.32 |
|  | 4300.0 | 3.63 | 0 | 10 | 10 | 1.0 | 5.60 | 0.52 |
|  | 430.0 | 2.63 | 0 | 10 | 10 | 1.0 | 6.20 | 0.63 |
|  | 43.0 | 1.63 | 0 | 10 | 10 | 1.0 | 6.60 | 0.70 |
|  | 4.3 | 0.63 | 5 | 5 | 10 | 0.5 | 7.20 | 0.28 |
|  | 0.4 | -0.37 | 10 | 0 | 10 | 0.0 | - | - |
| ΔFTT1597 | 10600.0 | 4.03 | 0 | 10 | 10 | 1.0 | 4.30 | 0.48 |
|  | 1060.0 | 3.03 | 0 | 10 | 10 | 1.0 | 4.70 | 0.48 |
|  | 106.0 | 2.03 | 5 | 5 | 10 | 0.5 | 6.20 | 1.10 |
|  | 12.66 | 1.10 | 7 | 3 | 10 | 0.3 | 7.00 | 0.00 |
|  | 0.74 | -0.13 | 9 | 1 | 10 | 0.1 | 11.00 | - |
|  | 0.06 | -1.22 | 10 | 0 | 10 | 0.0 | - | - |

We evaluated the effect of five mutants on *F. tularensis* virulence using mouse intranasal model experiments. As a positive control, we used the fully virulent wild-type *F. tularensis* subsp. *tularensis* Schu S4 strain. A total of 360 BALB/c mice [10 mice for each of six doses of colony-forming units (CFUs) for each of the 6 strains (5 mutant strains + the wild-type strain)] were exposed to different intranasal doses ranging from ≥0.03 CFU to ≥78,000 CFU in 10-fold increments and monitored for 21 days. We used the dose-response experiments to estimate the lethal dose for each strain.
