# Supplementary material for: Using host-pathogen protein interactions to identify and characterize Francisella tularensis virulence factors
Source: BMC Genomics. 2015 Dec 29;16:1106. doi: 10.1186/s12864-015-2351-1 (PMC4696196; doi:10.1186/s12864-015-2351-1)

**Figure S1.** Evaluation of the effect of five mutants on *Francisella tularensis* virulence using mouse intranasal model experiments. As a positive control, we used the fully virulent wild-type *F. tularensis* subsp. *tularensis* Schu S4 strain. A total of 360 BALB/c mice [10 mice for each of six doses of colony-forming units (CFUs) for each of the six strains (five mutant strains + the wild-type strain)] were exposed to intranasal doses ranging from ≥0.03 CFU to ≥78,000 CFU in 10-fold increments and monitored for 21 days.


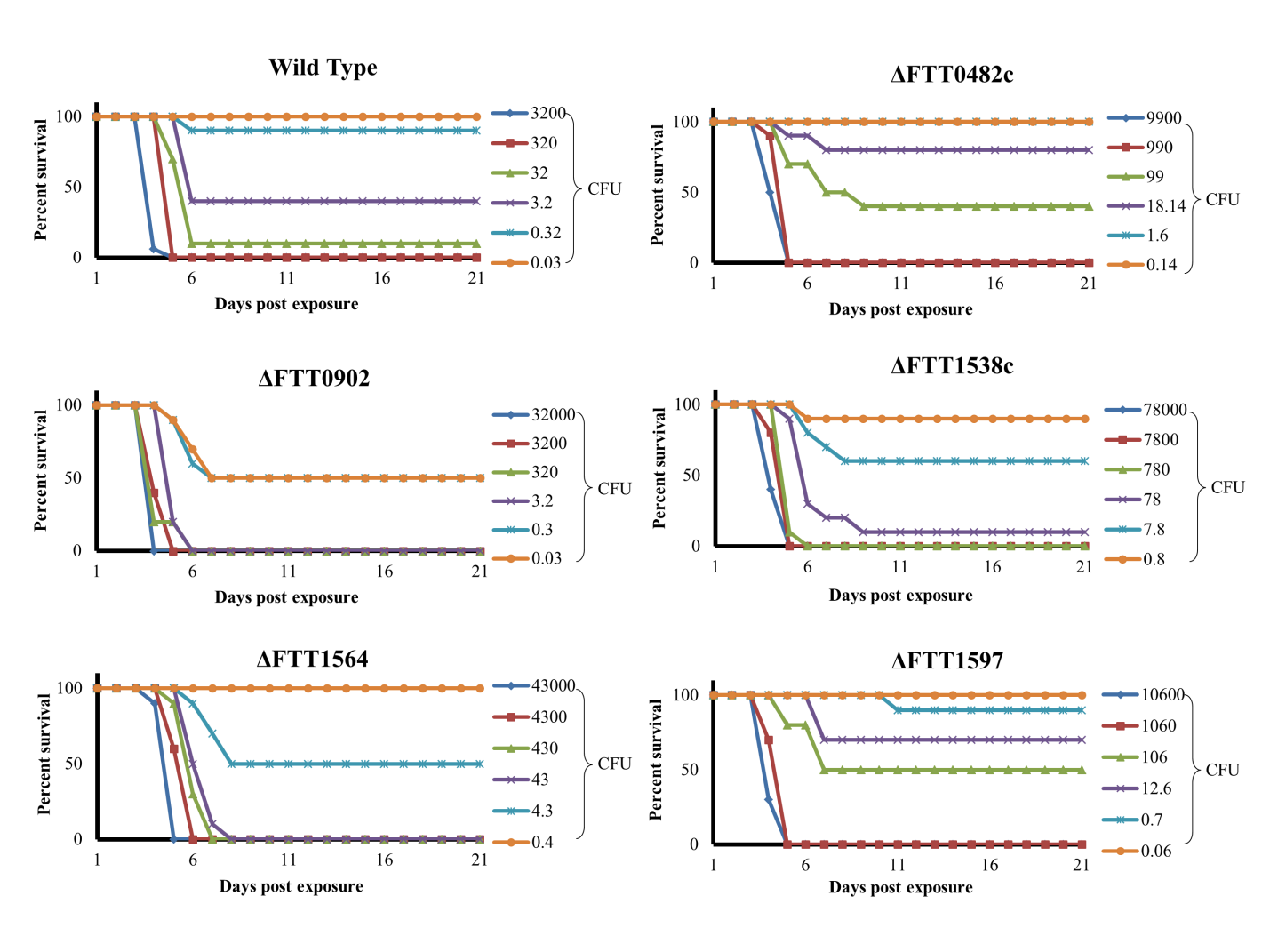

Supplement: Additional file 5: Figure S1. — Evaluation of the effect of five mutants on Francisella tularensis virulence using mouse intranasal model experiments. (DOCX 372 kb) [file 12864_2015_2351_MOESM5_ESM.docx]
