# Supplementary material for: Using host-pathogen protein interactions to identify and characterize Francisella tularensis virulence factors
Source: BMC Genomics. 2015 Dec 29;16:1106. doi: 10.1186/s12864-015-2351-1 (PMC4696196; doi:10.1186/s12864-015-2351-1)
Supplement: Additional file 6: Table S4. — Likelihood that one strain is more or less potent than any other strain. (DOCX 18 kb) [file 12864_2015_2351_MOESM6_ESM.docx]

**Table S4.** *Likelihood that one strain is more or less potent than any other strain*

|  | Δ**FTT0482c** | Δ**FTT0902** | Δ**FTT1538** | Δ**FTT1564** | Δ**FTT1597** | **Wild type** |
| --- | --- | --- | --- | --- | --- | --- |
| Δ**FTT0482c** | - | 0.00 | 0.00 | 0.00 | 0.25 | 0.00 |
| Δ**FTT0902** | 1.00 | - | 1.00 | 1.00 | 1.00 | 1.00 |
| Δ**FTT1538** | 1.00 | 0.00 | - | 0.06 | 0.98 | 0.03 |
| Δ**FTT1564** | 1.00 | 0.00 | 0.94 | - | 1.00 | 0.15 |
| Δ**FTT1597** | 0.75 | 0.00 | 0.02 | 0.00 | - | 0.00 |
| **Wild type** | 1.00 | 0.00 | 0.97 | 0.85 | 1.00 | - |

Comparison of the posterior samples of the 50% lethal dose (LD_50_) value of each strain allowed us to make probabilistic statements about how likely it is that one strain is more or less potent than any other strain at the median lethality level. Each row corresponds to one of the strains (five mutant strains and the wild-type strain), and values in each row correspond to the probability that a given strain has a LD_50_ value smaller than the LD_50_ values of the strain given in the column.
