# Supplementary material for: Using host-pathogen protein interactions to identify and characterize Francisella tularensis virulence factors
Source: BMC Genomics. 2015 Dec 29;16:1106. doi: 10.1186/s12864-015-2351-1 (PMC4696196; doi:10.1186/s12864-015-2351-1)
Supplement: Additional file 7: Table S5. — Human-F. tularensis protein-protein interactions retested in pairwise Y2H experiments. (DOCX 20 kb) [file 12864_2015_2351_MOESM7_ESM.docx]

**Table S5.** *Human-F. tularensis protein-protein interactions retested in pairwise Y2H experiments*

| ***F. tularensis* Protein** | **Human Protein** | **Human Protein Description** | **Pairwise Y2H** | **High-Throughput Y2H** |
| --- | --- | --- | --- | --- |
| FTT0482c | WDR48 | WD repeat-containing protein 48 | Positive | Positive |
| FTT0482c | EXOC1 | Exocyst complex component 1 | Negative | Positive |
| FTT0482c | TMEM167A | Protein kish-A | Negative | Positive |
| FTT0482c | NAPG | Gamma-soluble NSF attachment protein | Negative | Positive |
| FTT0482c | QKI | Protein quaking | Negative | Positive |
| FTT1538c | HSPA5 | 78 kDa glucose-regulated protein | Positive | Positive |
| FTT1538c | WDR48 | WD repeat-containing protein 48 | Positive | Positive |
| FTT1538c | STX8 | Syntaxin-8 | Negative | Positive |
| FTT1538c | PI4K2B | Phosphatidylinositol 4-kinase type 2-beta | Negative | Positive |
| FTT1538c | ARHGDIB | Rho GDP-dissociation inhibitor 2 | Negative | Positive |
| FTT1538c | AP3M1 | AP-3 complex subunit mu-1 | Positive | Negative |
| FTT1597 | AP3M1 | AP-3 complex subunit mu-1 | Positive | Positive |
| FTT1597 | PTPN6 | Tyrosine-protein phosphatase non-receptor type 6 | Negative | Positive |
| FTT1597 | WDR48 | WD repeat-containing protein 48 | Positive | Negative |
| FTT1597 | QKI | Protein quaking | Positive | Negative |

Y2H, yeast two-hybrid.
