# Supplementary material for: Using host-pathogen protein interactions to identify and characterize Francisella tularensis virulence factors
Source: BMC Genomics. 2015 Dec 29;16:1106. doi: 10.1186/s12864-015-2351-1 (PMC4696196; doi:10.1186/s12864-015-2351-1)
Supplement: Additional file 8: Table S6. — Enrichment of GO biological processes in host subnetworks. (DOCX 63 kb) [file 12864_2015_2351_MOESM8_ESM.docx]

**Table S6.** *Enrichment of GO biological processes in host subnetworks*

| **Term** | | | | **Size** | | | **p-value** | | | | |
| --- | --- | --- | --- | --- | --- | --- | --- | --- | --- | --- | --- |
| **ID** | | | **Description** | ***N*_C_** | ***N*_M_** | | **p_GO_** | | **p_Rp_** | | **p_Rn_** |
| GO:0008152 | | Metabolic process | 37 | 22 | | 2.0∙10^-4^ | | 0 | | 4.8∙10^-6^ |  |
| GO:0044237 | | Cellular metabolic process | 36 | 21 | | 1.0∙10^-4^ | | 0 | | 1.5∙10^-5^ |  |
| GO:0044238 | | Primary metabolic process | 35 | 20 | | 5.0∙10^-4^ | | 0 | | 2.8∙10^-5^ |  |
| GO:0044260 | | Cellular macromolecule metabolic process | 34 | 19 | | 0 | | 0 | | 6.9∙10^-5^ |  |
| GO:0043170 | | Macromolecule metabolic process | 34 | 19 | | 0 | | 0 | | 6.9∙10^-5^ |  |
| GO:0034641 | | Cellular nitrogen compound metabolic process | 30 | 15 | | 0 | | 0 | | 1.1∙10^-5^ |  |
| GO:0006807 | | Nitrogen compound metabolic process | 31 | 15 | | 0 | | 0 | | 8.8∙10^-5^ |  |
| GO:0006139 | | Nucleobase-containing compound metabolic process | 29 | 15 | | 0 | | 0 | | 1.0∙10^-5^ |  |
| GO:0044267 | | Cellular protein metabolic process | 22 | 13 | | 1.0∙10^-4^ | | 0 | | 8.0∙10^-8^ |  |
| GO:0090304 | | Nucleic acid metabolic process | 28 | 13 | | 0 | | 0 | | 2.7∙10^-5^ |  |
| GO:0019538 | | Protein metabolic process | 23 | 13 | | 2.0∙10^-4^ | | 0 | | 5.6∙10^-6^ |  |
| GO:0065007 | | Biological regulation | 37 | 12 | | 1.0∙10^-4^ | | 0 | | 2.1∙10^-3^ |  |
| GO:0010467 | | Gene expression | 26 | 12 | | 0 | | 0 | | 1.5∙10^-4^ |  |
| GO:0050789 | | Regulation of biological process | 37 | 12 | | 0 | | 0 | | 2.1∙10^-3^ |  |
| GO:0050794 | | Regulation of cellular process | 36 | 12 | | 0 | | 0 | | 2.0∙10^-3^ |  |
| GO:0016070 | | RNA metabolic process | 26 | 12 | | 0 | | 0 | | 3.8∙10^-5^ |  |
| GO:0031323 | | Regulation of cellular metabolic process | 32 | 11 | | 0 | | 0 | | 1.1∙10^-3^ |  |
| GO:0060255 | | Regulation of macromolecule metabolic process | 31 | 11 | | 0 | | 0 | | 1.1∙10^-3^ |  |
| GO:0019222 | | Regulation of metabolic process | 35 | 11 | | 0 | | 0 | | 2.5∙10^-3^ |  |
| GO:0080090 | | Regulation of primary metabolic process | 31 | 11 | | 0 | | 0 | | 1.1∙10^-3^ |  |
| GO:0006950 | | Response to stress | 18 | 10 | | 4.0∙10^-3^ | | 0 | | 4.5∙10^-4^ |  |
| GO:0006464 | | Cellular protein modification process | 18 | 10 | | 6.0∙10^-4^ | | 0 | | 8.0∙10^-8^ |  |
| GO:0043412 | | Macromolecule modification | 18 | 10 | | 7.0∙10^-4^ | | 0 | | 8.0∙10^-8^ |  |
| GO:0036211 | | Protein modification process | 18 | 10 | | 6.0∙10^-4^ | | 0 | | 8.0∙10^-8^ |  |
| GO:0065007 | | Biological regulation | 37 | 9 | | 1.0∙10^-4^ | | 2.0∙10^-4^ | | 4.0∙10^-3^ |  |
| GO:0044249 | | Cellular biosynthetic process | 26 | 9 | | 0 | | 1.0∙10^-4^ | | 9.9∙10^-4^ |  |
| **Term** | | | | **Size** | | | **p-value** | | | | |
| **ID** | | **Description** | | ***N*_C_** | ***N*_M_** | | **p_GO_** | | | **p_Rp_** | **p_Rn_** |
| GO:0009058 | Biosynthetic process | | 26 | 9 | | 1.0∙10^-4^ | | | 1.0∙10^-4^ | 9.9∙10^-4^ |  |
| GO:0034645 | Cellular macromolecule biosynthetic process | | 25 | 9 | | 0 | | | 0 | 9.3∙10^-4^ |  |
| GO:0009059 | Macromolecule biosynthetic process | | 25 | 9 | | 0 | | | 0 | 9.3∙10^-4^ |  |
| GO:0048519 | Negative regulation of biological process | | 23 | 9 | | 0 | | | 0 | 1.0∙10^-3^ |  |
| GO:0048523 | Negative regulation of cellular process | | 20 | 9 | | 0 | | | 0 | 1.3∙10^-4^ |  |
| GO:0050789 | Regulation of biological process | | 37 | 9 | | 0 | | | 2.0∙10^-4^ | 4.0∙10^-3^ |  |
| GO:0009889 | Regulation of biosynthetic process | | 26 | 9 | | 0 | | | 0 | 9.9∙10^-4^ |  |
| GO:0031326 | Regulation of cellular biosynthetic process | | 26 | 9 | | 0 | | | 0 | 9.9∙10^-4^ |  |
| GO:2000112 | Regulation of cellular macromolecule biosynthetic process | | 24 | 9 | | 0 | | | 0 | 8.3∙10^-4^ |  |
| GO:0050794 | Regulation of cellular process | | 36 | 9 | | 0 | | | 2.0∙10^-4^ | 4.0∙10^-3^ |  |
| GO:0010468 | Regulation of gene expression | | 24 | 9 | | 0 | | | 0 | 7.3∙10^-4^ |  |
| GO:0010556 | Regulation of macromolecule biosynthetic process | | 24 | 9 | | 0 | | | 0 | 8.3∙10^-4^ |  |
| GO:0051171 | Regulation of nitrogen compound metabolic process | | 25 | 9 | | 0 | | | 0 | 3.1∙10^-4^ |  |
| GO:0019219 | Regulation of nucleobase-containing compound metabolic process | | 25 | 9 | | 0 | | | 0 | 3.1∙10^-4^ |  |
| GO:2001141 | Regulation of RNA biosynthetic process | | 22 | 9 | | 0 | | | 0 | 2.2∙10^-4^ |  |
| GO:0051252 | Regulation of RNA metabolic process | | 23 | 9 | | 0 | | | 0 | 2.3∙10^-4^ |  |
| GO:0006355 | Regulation of transcription, DNA-dependent | | 21 | 9 | | 0 | | | 0 | 1.8∙10^-4^ |  |
| GO:0032774 | RNA biosynthetic process | | 22 | 9 | | 0 | | | 0 | 2.2∙10^-4^ |  |
| GO:0006351 | Transcription, DNA-dependent | | 22 | 9 | | 0 | | | 0 | 2.2∙10^-4^ |  |
| GO:0019222 | Regulation of metabolic process | | 35 | 8 | | 0 | | | 2.0∙10^-4^ | 4.6∙10^-3^ |  |
| GO:0042221 | Response to chemical stimulus | | 18 | 8 | | 7.0∙10^-4^ | | | 0 | 4.9∙10^-4^ |  |
| GO:0010033 | Response to organic substance | | 17 | 8 | | 0 | | | 0 | 4.6∙10^-4^ |  |
| GO:0023052 | Signaling | | 22 | 8 | | 9.2∙10^-3^ | | | 0 | 1.1∙10^-3^ |  |
| GO:0065007 | Biological regulation | | 37 | 7 | | 1.0∙10^-4^ | | | 6.0∙10^-4^ | 5.8∙10^-3^ |  |
| GO:0044260 | Cellular macromolecule metabolic process | | 34 | 7 | | 0 | | | 8.0∙10^-4^ | 5.6∙10^-3^ |  |
| GO:0044237 | Cellular metabolic process | | 36 | 7 | | 1.0∙10^-4^ | | | 1.2∙10^-3^ | 5.7∙10^-3^ |  |
| GO:0034641 | Cellular nitrogen compound metabolic process | | 30 | 7 | | 0 | | | 4.0∙10^-4^ | 1.9∙10^-3^ |  |
| GO:0070887 | Cellular response to chemical stimulus | | 17 | 7 | | 0 | | | 0 | 4.0∙10^-4^ |  |
| GO:0010467 | Gene expression | | 26 | 7 | | 0 | | | 1.0∙10^-4^ | 2.2∙10^-3^ |  |
| GO:0043170 | Macromolecule metabolic process | | 34 | 7 | | 0 | | | 9.0∙10^-4^ | 5.6∙10^-3^ |  |
| GO:0008152 | Metabolic process | | 37 | 7 | | 2.0∙10^-4^ | | | 1.4∙10^-3^ | 5.7∙10^-3^ |  |
| GO:0006807 | Nitrogen compound metabolic process | | 31 | 7 | | 0 | | | 4.0∙10^-4^ | 3.4∙10^-3^ |  |
| GO:0090304 | Nucleic acid metabolic process | | 28 | 7 | | 0 | | | 2.0∙10^-4^ | 1.4∙10^-3^ |  |
| GO:0006139 | Nucleobase-containing compound metabolic process | | 29 | 7 | | 0 | | | 3.0∙10^-4^ | 1.9∙10^-3^ |  |
| GO:0048518 | Positive regulation of biological process | | 21 | 7 | | 5.0∙10^-4^ | | | 1.0∙10^-4^ | 9.7∙10^-4^ |  |
| GO:0048522 | Positive regulation of cellular process | | 19 | 7 | | 1.2∙10^-3^ | | | 0 | 2.6∙10^-4^ |  |
| GO:0044238 | Primary metabolic process | | 35 | 7 | | 5.0∙10^-4^ | | | 1.3∙10^-3^ | 5.6∙10^-3^ |  |
| GO:0050789 | Regulation of biological process | | 37 | 7 | | 0 | | | 6.0∙10^-4^ | 5.8∙10^-3^ |  |
| GO:0031323 | Regulation of cellular metabolic process | | 32 | 7 | | 0 | | | 4.0∙10^-4^ | 3.9∙10^-3^ |  |
| GO:0050794 | Regulation of cellular process | | 36 | 7 | | 0 | | | 6.0∙10^-4^ | 5.7∙10^-3^ |  |
| GO:0010468 | Regulation of gene expression | | 24 | 7 | | 0 | | | 0 | 1.9∙10^-3^ |  |
| GO:0060255 | Regulation of macromolecule metabolic process | | 31 | 7 | | 0 | | | 3.0∙10^-4^ | 3.8∙10^-3^ |  |
| GO:0019222 | Regulation of metabolic process | | 35 | 7 | | 0 | | | 4.0∙10^-4^ | 5.6∙10^-3^ |  |
| GO:0080090 | Regulation of primary metabolic process | | 31 | 7 | | 0 | | | 3.0∙10^-4^ | 3.8∙10^-3^ |  |
| GO:0016070 | RNA metabolic process | | 26 | 7 | | 0 | | | 0 | 1.2∙10^-3^ |  |
| GO:0009058 | Biosynthetic process | | 26 | 6 | | 1.0∙10^-4^ | | | 6.0∙10^-4^ | 3.3∙10^-3^ |  |
| GO:0044249 | Cellular biosynthetic process | | 26 | 6 | | 0 | | | 6.0∙10^-4^ | 3.3∙10^-3^ |  |
| GO:0034645 | Cellular macromolecule biosynthetic process | | 25 | 6 | | 0 | | | 6.0∙10^-4^ | 3.2∙10^-3^ |  |
| GO:0009059 | Macromolecule biosynthetic process | | 25 | 6 | | 0 | | | 6.0∙10^-4^ | 3.2∙10^-3^ |  |
| **Term** | | | | **Size** | | | | **p-value** | | | |
| **ID** | | **Description** | | ***N*_C_** | ***N*_M_** | | | **p_GO_** | | **p_Rp_** | **p_Rn_** |
| GO:0007275 | Multicellular organismal development | | 19 | 6 | 9.2∙10^-3^ | | | | 1.0∙10^-4^ | 1.4∙10^-3^ |  |
| GO:0009890 | Negative regulation of biosynthetic process | | 11 | 6 | 1.0∙10^-4^ | | | | 0 | 1.3∙10^-4^ |  |
| GO:0031327 | Negative regulation of cellular biosynthetic process | | 11 | 6 | 1.0∙10^-4^ | | | | 0 | 1.3∙10^-4^ |  |
| GO:2000113 | Negative regulation of cellular macromolecule biosynthetic process | | 11 | 6 | 1.0∙10^-4^ | | | | 0 | 1.3∙10^-4^ |  |
| GO:0031324 | Negative regulation of cellular metabolic process | | 14 | 6 | 0 | | | | 0 | 4.1∙10^-4^ |  |
| GO:0010629 | Negative regulation of gene expression | | 12 | 6 | 0 | | | | 0 | 2.1∙10^-4^ |  |
| GO:0010558 | Negative regulation of macromolecule biosynthetic process | | 11 | 6 | 1.0∙10^-4^ | | | | 0 | 1.3∙10^-4^ |  |
| GO:0010605 | Negative regulation of macromolecule metabolic process | | 16 | 6 | 0 | | | | 0 | 9.2∙10^-4^ |  |
| GO:0009892 | Negative regulation of metabolic process | | 16 | 6 | 0 | | | | 0 | 9.2∙10^-4^ |  |
| GO:0051172 | Negative regulation of nitrogen compound metabolic process | | 12 | 6 | 0 | | | | 0 | 1.4∙10^-4^ |  |
| GO:0045934 | Negative regulation of nucleobase-containing compound metabolic process | | 12 | 6 | 0 | | | | 0 | 1.4∙10^-4^ |  |
| GO:0051253 | Negative regulation of RNA metabolic process | | 12 | 6 | 0 | | | | 0 | 1.4∙10^-4^ |  |
| GO:0045892 | Negative regulation of transcription, DNA-dependent | | 11 | 6 | 0 | | | | 0 | 1.3∙10^-4^ |  |
| GO:0031325 | Positive regulation of cellular metabolic process | | 15 | 6 | 4.0∙10^-4^ | | | | 0 | 1.0∙10^-4^ |  |
| GO:0010604 | Positive regulation of macromolecule metabolic process | | 16 | 6 | 1.0∙10^-4^ | | | | 0 | 1.4∙10^-4^ |  |
| GO:0009893 | Positive regulation of metabolic process | | 16 | 6 | 2.0∙10^-4^ | | | | 0 | 1.4∙10^-4^ |  |
| GO:0065008 | Regulation of biological quality | | 16 | 6 | 2.3∙10^-3^ | | | | 0 | 2.1∙10^-3^ |  |
| GO:0009889 | Regulation of biosynthetic process | | 26 | 6 | 0 | | | | 2.0∙10^-4^ | 3.3∙10^-3^ |  |
| GO:0031326 | Regulation of cellular biosynthetic process | | 26 | 6 | 0 | | | | 2.0∙10^-4^ | 3.3∙10^-3^ |  |
| GO:2000112 | Regulation of cellular macromolecule biosynthetic process | | 24 | 6 | 0 | | | | 2.0∙10^-4^ | 3.0∙10^-3^ |  |
| GO:0031323 | Regulation of cellular metabolic process | | 32 | 6 | 0 | | | | 5.0∙10^-4^ | 5.1∙10^-3^ |  |
| GO:0010556 | Regulation of macromolecule biosynthetic process | | 24 | 6 | 0 | | | | 2.0∙10^-4^ | 3.0∙10^-3^ |  |
| GO:0060255 | Regulation of macromolecule metabolic process | | 31 | 6 | 0 | | | | 5.0∙10^-4^ | 5.0∙10^-3^ |  |
| GO:0065009 | Regulation of molecular function | | 16 | 6 | 1.0∙10^-4^ | | | | 0 | 8.5∙10^-4^ |  |
| GO:0080090 | Regulation of primary metabolic process | | 31 | 6 | 0 | | | | 4.0∙10^-4^ | 5.0∙10^-3^ |  |
| GO:0007166 | Cell surface receptor signaling pathway | | 15 | 5 | 3.4∙10^-3^ | | | | 6.0∙10^-4^ | 1.8∙10^-3^ |  |
| GO:0016310 | Phosphorylation | | 10 | 5 | 9.7∙10^-3^ | | | | 2.0∙10^-4^ | 1.1∙10^-4^ |  |
| GO:0043085 | Positive regulation of catalytic activity | | 12 | 5 | 0 | | | | 0 | 2.6∙10^-4^ |  |
| GO:0051345 | Positive regulation of hydrolase activity | | 8 | 5 | 4.0∙10^-4^ | | | | 0 | 7.7∙10^-5^ |  |
| GO:0044093 | Positive regulation of molecular function | | 13 | 5 | 0 | | | | 0 | 6.3∙10^-4^ |  |
| GO:0006468 | Protein phosphorylation | | 10 | 5 | 4.2∙10^-3^ | | | | 1.0∙10^-4^ | 1.1∙10^-4^ |  |
| GO:0050790 | Regulation of catalytic activity | | 13 | 5 | 9.0∙10^-4^ | | | | 1.0∙10^-4^ | 5.5∙10^-4^ |  |
| GO:0032268 | Regulation of cellular protein metabolic process | | 12 | 5 | 7.0∙10^-4^ | | | | 3.0∙10^-4^ | 5.6∙10^-4^ |  |
| GO:0050793 | Regulation of developmental process | | 11 | 5 | 2.9∙10^-3^ | | | | 0 | 2.6∙10^-4^ |  |
| GO:0051336 | Regulation of hydrolase activity | | 8 | 5 | 2.9∙10^-3^ | | | | 0 | 7.7∙10^-5^ |  |
| GO:0051171 | Regulation of nitrogen compound metabolic process | | 25 | 5 | 0 | | | | 1.1∙10^-3^ | 3.1∙10^-3^ |  |
| GO:0019219 | Regulation of nucleobase-containing compound metabolic process | | 25 | 5 | 0 | | | | 9.0∙10^-4^ | 3.1∙10^-3^ |  |
| GO:0042325 | Regulation of phosphorylation | | 8 | 5 | 9.6∙10^-3^ | | | | 0 | 5.7∙10^-5^ |  |
| GO:0051246 | Regulation of protein metabolic process | | 13 | 5 | 5.0∙10^-4^ | | | | 3.0∙10^-4^ | 9.2∙10^-4^ |  |
| GO:0031399 | Regulation of protein modification process | | 9 | 5 | 6.8∙10^-3^ | | | | 0 | 8.2∙10^-5^ |  |
| GO:0001932 | Regulation of protein phosphorylation | | 8 | 5 | 6.6∙10^-3^ | | | | 0 | 5.7∙10^-5^ |  |
| GO:2001141 | Regulation of RNA biosynthetic process | | 22 | 5 | 0 | | | | 6.0∙10^-4^ | 2.8∙10^-3^ |  |
| GO:0051252 | Regulation of RNA metabolic process | | 23 | 5 | 0 | | | | 6.0∙10^-4^ | 2.8∙10^-3^ |  |
| GO:0006357 | Regulation of transcription from RNA polymerase II promoter | | 10 | 5 | 4.1∙10^-3^ | | | | 1.0∙10^-4^ | 1.1∙10^-4^ |  |
| GO:0006355 | Regulation of transcription, DNA-dependent | | 21 | 5 | 0 | | | | 6.0∙10^-4^ | 2.6∙10^-3^ |  |
| GO:0032774 | RNA biosynthetic process | | 22 | 5 | 0 | | | | 7.0∙10^-4^ | 2.8∙10^-3^ |  |
| GO:0023052 | Signaling | | 22 | 5 | 9.2∙10^-3^ | | | | 2.2∙10^-3^ | 4.1∙10^-3^ |  |

| **Term** | | | **Size** | | **p-value** | | |
| --- | --- | --- | --- | --- | --- | --- | --- |
| **ID** | | **Description** | ***N*_C_** | ***N*_M_** | **p_GO_** | **p_Rp_** | **p_Rn_** |
| GO:0006366 | Transcription from RNA polymerase II promoter | 14 | 5 | 1.0∙10^-4^ | 1.0∙10^-4^ | 2.9∙10^-4^ |  |
| GO:0006351 | Transcription, DNA-dependent | 22 | 5 | 0 | 6.0∙10^-4^ | 2.8∙10^-3^ |  |
| GO:0007049 | Cell cycle | 11 | 4 | 3.3∙10^-3^ | 1.7∙10^-3^ | 2.0∙10^-3^ |  |
| GO:0003006 | Developmental process involved in reproduction | 6 | 4 | 1.5∙10^-3^ | 0 | 2.5∙10^-5^ |  |
| GO:0007275 | Multicellular organismal development | 19 | 4 | 9.2∙10^-3^ | 2.7∙10^-3^ | 4.4∙10^-3^ |  |
| GO:0044092 | Negative regulation of molecular function | 8 | 4 | 1.7∙10^-3^ | 1.0∙10^-4^ | 1.5∙10^-3^ |  |
| GO:0051173 | Positive regulation of nitrogen compound metabolic process | 10 | 4 | 2.7∙10^-3^ | 3.0∙10^-4^ | 3.6∙10^-4^ |  |
| GO:0045935 | Positive regulation of nucleobase-containing compound metabolic process | 10 | 4 | 2.4∙10^-3^ | 3.0∙10^-4^ | 3.6∙10^-4^ |  |
| GO:0045944 | Positive regulation of transcription from RNA polymerase II promoter | 7 | 4 | 6.4∙10^-3^ | 1.0∙10^-4^ | 1.5∙10^-4^ |  |
| GO:0032446 | Protein modification by small protein conjugation | 9 | 4 | 0 | 1.0∙10^-4^ | 2.9∙10^-5^ |  |
| GO:0070647 | Protein modification by small protein conjugation or removal | 9 | 4 | 1.0∙10^-4^ | 1.0∙10^-4^ | 2.9∙10^-5^ |  |
| GO:0016567 | Protein ubiquitination | 9 | 4 | 0 | 1.0∙10^-4^ | 2.9∙10^-5^ |  |
| GO:0051726 | Regulation of cell cycle | 9 | 4 | 6.0∙10^-4^ | 7.0∙10^-4^ | 1.1∙10^-3^ |  |
| GO:2000026 | Regulation of multicellular organismal development | 10 | 4 | 1.4∙10^-3^ | 3.0∙10^-4^ | 4.5∙10^-4^ |  |
| GO:0051090 | Regulation of sequence-specific DNA binding transcription factor activity | 6 | 4 | 1.1∙10^-3^ | 0 | 1.1∙10^-4^ |  |
| GO:0009611 | Response to wounding | 9 | 4 | 8.3∙10^-3^ | 4.0∙10^-4^ | 1.3∙10^-3^ |  |
| GO:0006366 | Transcription from RNA polymerase II promoter | 14 | 4 | 1.0∙10^-4^ | 9.0∙10^-4^ | 9.0∙10^-4^ |  |

*N*_C_, number of proteins in the largest connected component annotated with a given term; *N*_M_, number of proteins in the largest interaction module for a given term; p_GO_, probability of the same number of proteins as the largest connected component being annotated with a given Gene Ontology (GO) term solely through a random selection; p_Rn_, probability that a given number of proteins as *N*_M_ are annotated with a given GO term solely through random selection in a random network that has the same degree distribution as our human network; p_Rp_, probability that a given number of proteins as *N*_M_ are annotated with a given GO term solely through random selection. This table contains only the largest statistically significant interaction module for each term.
